# Biogeographic consequences of shifting climate for the western massasauga (*Sistrurus tergeminus*)

**DOI:** 10.1002/ece3.8599

**Published:** 2022-02-10

**Authors:** Danielle K. Walkup, Anna Michelle Lawing, Toby J. Hibbitts, Wade A. Ryberg

**Affiliations:** ^1^ Texas A&M Natural Resources Institute College Station Texas USA; ^2^ 14736 Department of Ecology and Conservation Biology Texas A&M University College Station Texas USA; ^3^ 14736 Biodiversity Research and Teaching Collection Department of Ecology and Conservation Biology Texas A&M University College Station Texas USA

**Keywords:** ecological niche models, forecasting, hindcasting, paleoclimate, paleogeography, potential geographic distribution, *Sistrurus tergeminus*, western massasauga

## Abstract

The western massasauga (*Sistrurus tergeminus*) is a small pit viper with an extensive geographic range, yet observations of this species are relatively rare. They persist in patchy and isolated populations, threatened by habitat destruction and fragmentation, mortality from vehicle collisions, and deliberate extermination. Changing climates may pose an additional stressor on the survival of isolated populations. Here, we evaluate historic, modern, and future geographic projections of suitable climate for *S. tergeminus* to outline shifts in their potential geographic distribution and inform current and future management. We used maximum entropy modeling to build multiple models of the potential geographic distribution of *S. tergeminus*. We evaluated the influence of five key decisions made during the modeling process on the resulting geographic projections of the potential distribution, allowing us to identify areas of model robustness and uncertainty. We evaluated models with the area under the receiver operating curve and true skill statistic. We retained 16 models to project both in the past and future multiple general circulation models. At the last glacial maximum, the potential geographic distribution associated with *S. tergeminus* occurrences had a stronghold in the southern part of its current range and extended further south into Mexico, but by the mid‐Holocene, its modeled potential distribution was similar to its present‐day potential distribution. Under future model projections, the potential distribution of *S. tergeminus* moves north, with the strongest northward trends predicted under a climate scenario increase of 8.5 W/m^2^. Some southern populations of *S. tergeminus* have likely already been extirpated and will continue to be threatened by shifting availability of suitable climate, as they are already under threat from desertification of grasslands. Land use and habitat loss at the northern edge of the species range are likely to make it challenging for this species to track suitable climates northward over time.

## Graphical abstract

The western massasauga (*Sistrurus tergeminus*) is a small pit viper with an extensive geographic range, yet they persist in patchy and isolated populations, threatened by habitat destruction and fragmentation, mortality from vehicle collisions, and deliberate extermination. Changing climates may pose an additional stressor on the survival of isolated populations. Here, we evaluate historic, modern, and future geographic projections of suitable climate for *S. tergeminus* to outline shifts in their potential geographic distribution and inform current and future management.

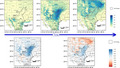

## 1 INTRODUCTION

The western massasauga (*Sistrurus tergeminus*; Say, 1823) is a small pit viper with an extensive geographic distribution in western North American grasslands, yet observations of this species are relatively rare. *Sistrurus tergeminus* is a species of conservation concern in Colorado (CPW, 2015) and Arizona (AZDGF, 2012) because large swaths of potentially suitable habitat have been converted to cropland, are degraded by conversion to grazing land, or are depauperate of prey populations due to water withdrawal for agriculture and other causes of xerification (Anderson et al., 2009; Mackessy, 2005; Ryberg et al., 2015). Although range maps are often depicted as continuous, the geographic range of this species is actually patchy and thought to be shaped by narrow ecological tolerances (Greene, 1994). Holocene climate changes may have left behind fragmented suitable habitat for this species (Greene, 1994, 1997). It is unclear whether the fragmented nature of the populations within this species is the result of converted and degraded land or a feature of the legacy of its climate history.

The legacy of climate history often shapes the current distribution of biodiversity (Dynesius & Jansson, 2000; Ricklefs & Schluter, 1993; Wiens & Donoghue, 2004). Increased availability of spatially explicit paleoclimatic models and data, along with enhanced molecular tools capable of testing more refined phylogeographic hypotheses, has made the investigation of the effects of climate history more readily available (Lawing, 2021; Svenning et al., 2015). Paleoclimatic legacies have important implications for biodiversity conservation as they identify (1) where species might experience climatically stable refugia worthy of long‐term protection (Ackerly et al., 2010; Loarie et al., 2009), and (2) which species may not be able to track climate changes via migration due to biogeographic constraints or human‐impacted areas (Bertrand et al., 2011; Lunt et al., 2013). Answering these questions for *S. tergeminus* is critical because of the fragmented nature of its populations and threats to its grassland habitat.

Recent phylogeographic research of *S. tergeminus* demonstrates that effective population sizes are large relative to time since divergence (Ryberg et al., 2015). There are eight well‐supported, equally divergent genetic clades, many of which lay on the margins of the geographic range of *S. tergeminus* (Ryberg et al., 2015). This species experienced a recent, rapid demographic expansion from a compact refugium, evidenced by a star‐like haplotype network (i.e., central ancestral haplotype surrounded by short branches depicting descendant haplotypes) (Slatkin & Hudson, 1991). Although low genetic diversity is expected at the periphery following such an expansion due to founder events and bottlenecks, substantial genetic diversity persists in the peripheral populations despite declining population census sizes (Anderson et al., 2009; Ryberg et al., 2015). This pattern of genetic diversity indicates that effective population sizes of *S. tergeminus* are still large and are likely preventing genetic drift from bringing loci to fixation (Maddison & Knowles, 2006; Ryberg et al., 2015).

One possible paleogeographic reconstruction based on the phylogenetic evidence described above is that *S. tergeminus* colonized much of its current range relatively recently from a single refugium as grasslands and desert thornscrub expanded at the end of the Last Glacial Maximum (LGM; Axelrod, 1985; Metcalfe, 2006). Holocene climate fluctuations causing expansion and contraction of grassland and thornscurb habitats then probably contributed to the recent divergences of peripheral *S. tergeminus* populations. However, this interpretation of phylogenetic evidence assumes complete sampling across both current and past (e.g., fossil) distributions of *S. tergeminus*. Although recent phylogenetic sampling efforts were robust (Ryberg et al., 2015), some notable gaps in the sampling distribution were apparent, namely from currently occupied habitats in northeastern Mexico and western Texas (e.g., central and northeast Chihuahuan Desert), and samples from those areas could influence relationships among clades. Furthermore, sampling across the fossil record for this species is extremely poor potentially obscuring evidence that geographical barriers did reduce gene flow and create subdivision in populations of the species that simply did not persist.

Here, we aim to evaluate historic, modern, and future potential geographic distributions of suitable climate and environment of *S. tergeminus* to identify shifts in available suitable climate and environment and to help inform current and future management. We estimate suitable climate and environment associated from *S. tergeminus* occurrences using ecological niche modeling methodology and hindcasts to the LGM and mid‐Holocene to highlight the fine‐grained spatial context for refugia and migration within the historic potential distribution of *S. tergeminus*. By studying past potential refugia, we hope to identify contemporary refugia and predict their potential conservation significance under the threat of a rapidly changing climate. Specifically, our objectives for this paper were to (1) determine important climatic and environmental influences across the distribution of *S. tergeminus*, (2) estimate the historic (LGM and mid‐Holocene) refugia of *S. tergeminus*, (3) draw inferences about their current distribution and genetic population structure, and (4) project the potential distribution under future climate change scenarios to pinpoint sites where the *S. tergeminus* is most at risk from changing climates. Our strategy for building ecological niche models was to be comprehensive and robust in attending to the many modeling decisions required for this approach. Thus, as an additional objective, we tested different settings and their effect on model performance to evaluate the optimal settings for our model, providing transparency in decision making and in the evaluation of the suitability of our models to inform conservation plans.

## 2 MATERIALS AND METHODS

We used maximum entropy to model the ecological niche and potential geographic distribution of *S. tergeminus*. We follow the conceptual framework and methodological terminology discussed by Peterson and Soberón (2012) and Peterson et al. (2011). We built multiple models of the ecological niche via climate and environmental predictors and projected the models onto multiple climate scenarios to fully explore the past, modern, and future potential geographic distribution of *S. tergeminus*. We evaluated the influence of five key decisions made during the modeling process on geographic projections of the potential distribution, which allowed us to identify areas of model robustness and uncertainty. Decisions included predictor variable selection, number of background points, shape of background polygon, bin size of environmental filters, and geographic bias in testing and training datasets. We used multiple evaluation statistics and projected models in the past and in the future using multiple climate scenarios. Sofaer et al. (2019) proposed a rubric for species distribution model developers, also applicable to the development of the closely related ecological niche models, to use to communicate model attributes and appropriate uses. We followed recommendations of Sofaer et al. (2019) to provide transparency in decision making and to evaluate the suitability of our models to inform conservation plans; we provide rubric assessment in Table S1.1. We deposited all R scripts and publicly available data on github (https://git.io/JL831) and other potentially sensitive localities are available on request. We scripted all models in R v. 3.6.1 (R Core Team, 2019).

### 2.1 Taxonomy and study area

Until recently, massasauga were grouped into three subspecies: *Sistrurus catenatus catenatus* (eastern massasauga), *S. c. tergeminus* (prairie massasauga), and *S. c. edwardsii* (desert massasauga). Kubatko et al. (2011) elevated *S. c. catenatus* to full species status, leaving *S. c. tergeminus* and *S. c. edwardsii* as subspecies. There has been ongoing confusion surrounding the localities of type specimens of these subspecies (Holycross et al., 2008), leaving the subsequent naming of the newly elevated species in question. However, in 2013, the International Commission on Zoological Nomenclature (ICZN) published a final ruling that formally split this rattlesnake into two full species (Crother et al., 2011, 2012; ICZN, 2013): the eastern massasauga (*S. catenatus*) and the western massasauga (*S. tergeminus*), with *S. tergeminus* remaining split into the two subspecies, *S. t. edwardsii* and *S. t. tergeminus*. More recently, *S. t. tergeminus* and *S. t. edwardsii* have been found to have low genetic differentiation (Ryberg et al., 2015) and Bylsma et al. (2021) have recommended that *S. tergeminus* be considered a single, genetically unified species.

**FIGURE 1:**
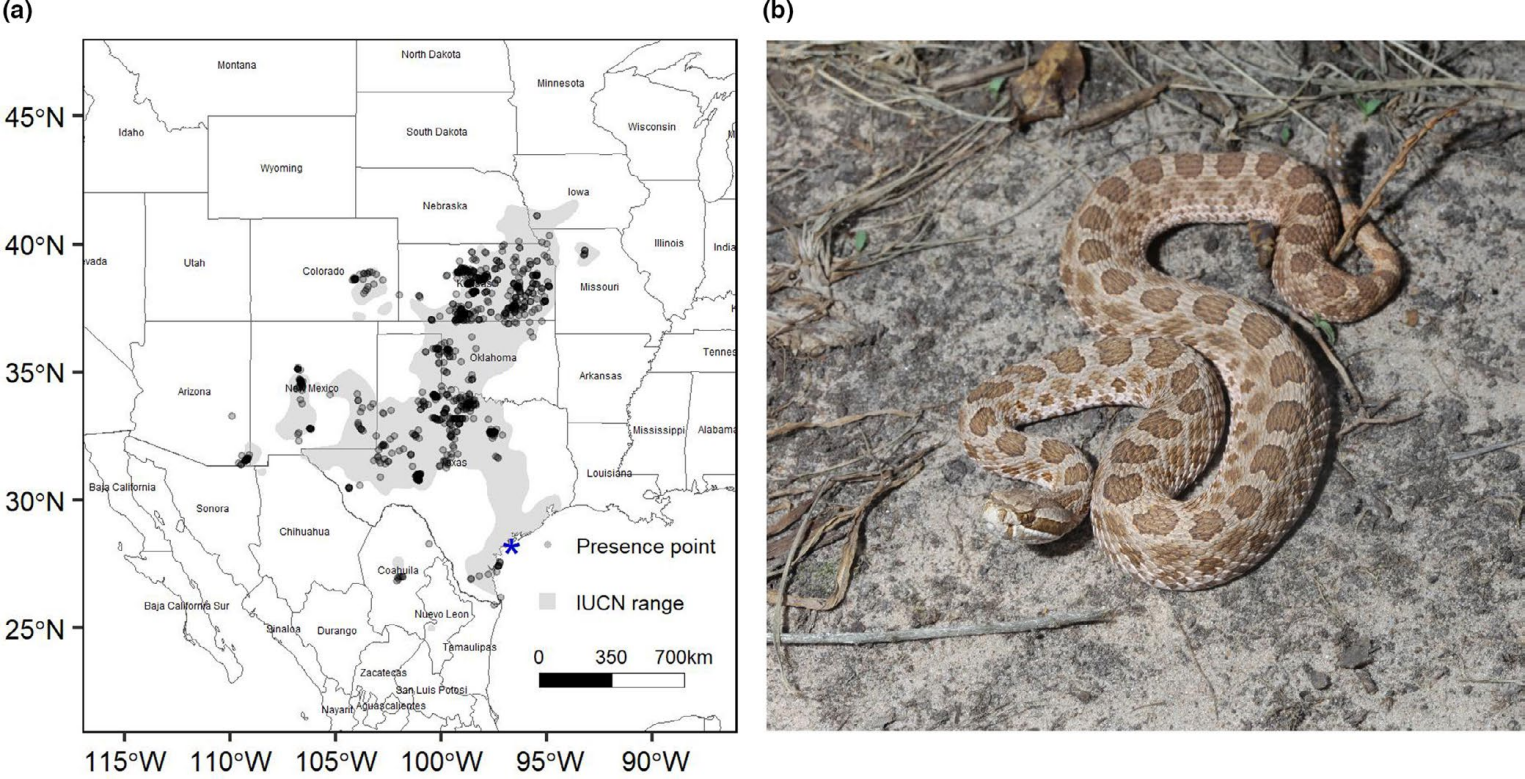
(a) Presence points for *Sistrurus tergeminus*, aggregated from natural history museum records, iNaturalist, and the Global Biodiversity Information Facility, overlaying the IUCN range (Frost et al., 2007). The points are a transparent gray, so the more points that aggregate in an area, the darker those points appear. The blue star indicates Matagorda and Padre Islands. (b) *Sistrurus tergeminus*, the study species

*Sistrurus tergeminus* ranges from the Tamaulipan Plains in Mexico north into the Great Plains of Nebraska, and west through New Mexico and Colorado, into the western edge of the Chihuahuan Desert in southeastern Arizona (Figure 1). The North American grassland biome that this species occupies stretches from mixed grass prairies of the Canadian Provinces of Alberta, Saskatchewan, and Manitoba to desert grasslands of the southwestern United States (US) and northern and central Mexico (Risser et al., 1981). The 25 degree span in latitude for the North American grassland biome is characterized by a gradient of mean annual temperatures ranging from 2.8°C in Regina, Canada settled within the northern mixed grass prairie to 22.6°C in Monterrey, Mexico at the edge of Chihuahuan Desert grasslands (Anderson, 2006). Precipitation systems shift from north to south in this biome with a greater percentage of annual precipitation falling in the winter around southern Canada, then in spring and summer throughout the central US to northern Texas, and finally summer monsoons and tropical storms contribute more to annual precipitation in the Chihuahuan Desert grasslands of the southwestern US and northern and central Mexico (Roy et al., 2019; Tang & Reiter, 1984). Changes in the magnitude and geographical ranges of these precipitation systems over millennia have altered both the overall availability and seasonal distribution of moisture and consequently, droughts, which has caused restricted extinctions and significantly influenced the evolutionary history of grassland plant and animal populations (Metcalfe et al., 1997; Steinauer & Collins, 1996).

Although sampling across the fossil record for this species is extremely poor, the few known fossils highlight relevant locations within the study area. A single *Sistrurus* fossil from the Pratt Slide in present‐day Nebraska places the genus in Miocene North America about 10–13 mya, two fossils from Kansas and Nebraska place it in the middle Pliocene, 2–4 mya, and one fossil from Kansas places the genus in the Pleistocene, 0.3–2 mya (Parmley & Hunter, 2010; Rogers, 1984). These records from the northern part of the current distribution suggest that *S. tergeminus* distribution contracted and expanded multiple times with each glacial cycle.

### 2.2 Paleobiogeographic setting

The distribution of grassland biodiversity, in particular, often reflects past changes in climate, as the current extent of the grassland biome globally has largely been determined by variation in past precipitation gradients (Anderson, 2006; Axelrod, 1985; Oesterheld et al., 1999). Although North American grasses have been present for at least 20 million years (Axelrod, 1985), their present distribution, and the biodiversity they contain, are relatively recent in origin (Anderson, 2006; Knopf & Samson, 1997). During the Pleistocene, repeated glacial advances caused a southward displacement of the mid‐latitude Westerlies bringing more winter rain to the mid‐continent and throughout the basin and range province (*sensu* Eaton, 1982; Parsons, 2006) possibly extending as far south as central Mexico (Metcalfe et al., 2000; Palacios et al., 2020). As a result, during the Last Glacial Maximum (LGM: 26,500–19,000 years ago) of the Pleistocene, for example, North American grasslands were drastically reduced (Bryson et al., 1970) and replaced or mixed with different communities of plants and animals depending on location (Dort & Jones, 1970; Ruddiman & Wright, 1987). The Great Plains from southern Canada through North and South Dakota to the Llano Estacado dotted with thousands of playa lakes in New Mexico and western Texas (Hafsten, 1961; Wendorf, 1961) was dominated by park‐like, open‐canopy coniferous woodlands with areas of open boreal spruce forest in the Central Plains of Kansas and Missouri extending eastward to the Appalachian Mountains (Wright et al., 1987). Although distant from North American continental glaciers, present‐day Chihuahuan Desert grasslands experienced equable climates, which lowered elevational and southern range limits of pinyon‐juniper‐oak woodlands without extirpating endemic desert scrub succulents and subtropical plants, resulting in vegetation assemblages that have no modern analogs (Van Devender, 1990).

When combined with the complex basin and range topography, these glacial cycles created periodic, southerly ecogeographic barriers to dispersal and climate refugia that have had different effects on the vertebrate taxa of southwestern North America (Knopf & Samson, 1997). Some small mammals, reptiles, and an amphibian that occupied this region before the LGM display strong phylogeographic structure associated with multiple Pleistocene refugia or ecogeographic barriers (small mammals: Andersen & Light, 2012; Neiswenter & Riddle, 2010, 2011; Riddle et al., 2000; Riddle & Hafner, 2006; reptiles and amphibians: Castoe et al., 2007; Douglas et al., 2006; McGuire et al., 2007; Pyron & Burbrink, 2010; Zamudio et al., 1997). In contrast, other taxa exhibit weak or shallow phylogeographic structure, a pattern consistent with a recent expansion from a single refugium (birds: Williford et al., 2013; Zink, 2002; Zink et al., 2001; small mammals: Riddle & Hafner, 2006; reptiles: Douglas et al., 2006). Within this setting, we investigated the paleogeographic distribution of a grassland‐dependent species, the western massasauga (*Sistrurus tergeminus*), in order to better understand current population distribution and genetic structure, as well as potential future risks from changing climates.

### 2.3 Occurrence data

We collected occurrence data from online databases, direct contact with collections, literature searches, and targeted field survey efforts. We queried the Global Biodiversity Information Facility (GBIF, accessed 20 June 2019) using package “rgbif” (Chamberlain et al., 2020), iNaturalist (accessed 13 June 2019, 16 November 2017, and 27 June 2017), and gathered specimen records from natural history collections using VertNet (accessed 20 June 2019) or through directly contacting collections with significant *S. tergeminus* holdings. These observations were distributed between 1903 and 2019, with the majority of the data being collected after the 1990s. Occurrences associated with fossils were found from the paleobiology database, PBDB (accessed 20 June 2019), and literature searches. Because of the differences in taxonomy updates across these different platforms, we queried GBIF using “*Sistrurus catenatus*”, iNaturalist using “*Sistrurus tergeminus*”, and the PBDB using “*Sistrurus*”.

Previous iterations of the modeled potential geographic distribution for this species were summarized in a report to New Mexico Department of Game and Fish (Ryberg et al., 2017). We used their model projections on maps of modern climate to focus survey effort in areas to try to fill in gaps in sampling (Ryberg et al., 2020). The current occurrence dataset filled gaps in Colorado and Missouri, and increased coverage throughout the geographic range. We removed records east of the Mississippi River to remove *S. catenatus*, *sensu stricto* (Kubatko et al., 2011). We also removed duplicate records and any records with recorded location uncertainty greater than 1 km. Occurrences were plotted and compared with the species’ known geographic range (Figure 1); any questionable outliers determined by a subject matter expert (TJH) were removed. This left us with a data set of 999 occurrences spanning the range of *S. tergeminus* (Figure 1a).

We filtered occurrence data to account for collection bias and bias from intensive sampling in readily accessible geographic areas (Boria et al., 2014; Varela et al., 2014). We chose to use environmental filtering, because it subsets environmental space, instead of geographic space, to account for intensely sampled areas. This method was shown to reduce bias and improve predictions of ecological niche models (Varela et al., 2014). Instead of using many climate variables within our environmental filter, we used the first four principal components axes of our combined climate variables to capture the four axes of greatest variation following Castellanos et al. (2019). We used the PC axes as the environmental space for the environmental filter. We binned the four PC axes with three bin sizes of 0.15, 0.3, and 0.75 bins and randomly selected one occurrence from within each bin, resulting in 336, 515, and 579 occurrence points, respectively.

### 2.4 Climate and environmental data

We evaluated climate and environmental variables for inclusion in our models from two data repositories, WorldClim and Envirem, based on the amount of variation captured by the variables and from expert knowledge of the ecology of the species. Tracy et al. (2018) found that machine learning approaches to variable selection were as good as expert selected variables based on the ecology of a species. Climate and environmental data were downloaded at 2.5’ resolution. There are 19 bioclim variables in the WorldClim repository and these variables were created using records spanning 1950–2000 (Hijmans et al., 2005). Bioclim variables are derived from monthly, quarterly, and annual summaries of daily weather records and are considered biologically meaningful descriptors of the climate (Nix, 1986). Bioclim variables represent the means and extremes of temperature and precipitation at three temporal scales (i.e., monthly, quarterly, and annual). There are 18 envirem variables in the envirem repository; derived from temperature, precipitation, and extraterrestrial solar radiation, covering the same time period as the bioclim variables (Title & Bemmels, 2018). Envirem variables include biologically relevant climate variables derived from monthly temperature, precipitation, and extraterrestrial solar radiation as well as two variables derived from digital elevation maps, all of which are intended to complement the bioclim variables set (Title & Bemmels, 2018). The 2.5’ resolution ensured that the spatial error of the occurrences (1 km) was smaller than the spatial grain of the model.

We overlaid the occurrence dataset with the climate and environmental raster datasets and extracted the values of all the variables geographically associated with each occurrence. From these 37 variables, we chose three different variable sets based on different types of criteria. For the first set, we used singular value decomposition in a principal components ordination to identify which variables had the highest or lowest loading on each of the first few PC axes, using the function prcomp from the “stats” package in R (R Core Team, 2019). The first four axes of the principal components ordination represented 91% of the independent orthogonal variation in the climate dataset. We narrowed the dataset to relatively uncorrelated variables that were highly loaded on the first four axes, and then species experts (TJH and WAR) narrowed it to 4 and 6 variables based on the ecology of the species. From the first four PC axes, we identified four variables that had low correlation values (*r* < |.321|) and that contributed to the most variation between species occurrences. This set includes temperature seasonality, maximum temperature of the warmest month, mean temperature of the wettest quarter, and precipitation of the coldest quarter (Table 1). For the second set, we kept the five variables that had the highest permutation importance out of all the 37 potential variables in the envirem and bioclim datasets and had medium correlation values (*r* < |.726|; Table 1). This set includes precipitation of the driest month, Thornthwaite aridity index, growing degree days (5℃), and potential evapotranspiration seasonality. Finally, for the third set, we combined the first two variable sets, removing any variables that were highly correlated (leaving a maximum *r* < |.726|) (Table 1). We removed temperature seasonality and precipitation of the coldest quarter because they were each highly correlated with other variables (*r* > |.809|).

**TABLE 1:** Descriptions of the climate variables used in the Maxent models to predict the likelihood of occurrence of *Sistrurus tergeminus*, from the envirem (Title & Bemmels, 2018) and bioclim (Hijmans et al., 2005) data sets

Data set	Code	Name	Definition	Variable set		
				1	2	3
Bioclim	Bio4	Temperature Seasonality	Standard deviation of the 12‐month average temperatures	✓	–	–
Bioclim	Bio5	Max Temperature of the Warmest Month	Maximum monthly temperature over a year	✓	–	✓
Bioclim	Bio8	Mean Temperature of the Wettest Quarter	Average temperature of the wettest 3‐month period	✓	–	✓
Bioclim	Bio14	Precipitation of the Driest Month	Total precipitation from the driest month	–	✓	✓
Bioclim	Bio19	Precipitation of the Coldest Quarter	Total rainfall for the coldest 3‐month period	✓	–	–
Envirem	Arid	Thornthwaite Aridity Index	Index of the degree of water deficit below water need	–	✓	✓
Envirem	GDD5	Growing Degree Days (5℃)	Sum of mean monthly temperature for months with mean temperature greater than 5℃ multiplied by number of days	–	✓	✓
Envirem	PETs	Potential Evapotranspiration seasonality	Monthly variability in potential evapotranspiration	–	✓	✓

Note

Variable set identifies which variables are used in each of the three climate variable sets used for prediction in the Maxent models.

### 2.5 Background points and extent

We sampled background points in eight ways, taking into account number of points, spatial extent, and sampling bias. These eight combinations were as follows, with definitions of each to follow: (1) 100 km radius point‐buffered extent with 1000 background points; (2) 100 km radius point‐buffered extent with 10,000 background points; (3) 200 km radius point‐buffered extent, 1000 background points; (4) 200 km radius point‐buffered extent, 10,000 background points; (5) minimum convex polygon (MCP) extent, 1000 points; (6) MCP extent, 10,000 background points; (7) buffered MCP extent, 1000 points; (8) buffered MCP extent, 10,000 points. We sampled at 1000 and 10,000 points (Barbet‐Massin et al., 2012; Phillips et al., 2009).

Background points drawn from too small or too large an area can result in spurious models or exaggerated statistical significance, so background points were distributed randomly within a Minimum Convex Polygon (MCP) around all the original occurrence points, and within a buffered MCP, adding 20% area to the extent (Barve et al., 2011; Jarnevich et al., 2017; Van Der Wal et al., 2009). Because sampling bias has been shown to result in biased estimation of environmental relationships, we created buffers with a radius of 100 km and 200 km around each point, merged each respective buffer into a polygon, and sampled random background points from the resulting polygons (Guillera‐Arroita et al., 2015). The 100 km buffer was a reasonable starting extent because it well encompassed known movement parameters for *S. tergeminus* (Patten et al., 2016; Wastell & MacKessy, 2011). The 200 km buffer accounted for a 76% increase in the background extent. These choices allowed us to mimic a bias of background points toward the actual presence points, but maintain our predictive power (Jarnevich et al., 2017; Van Der Wal et al., 2009). This mid‐high percentage buffer is reasonable, and should not overly inflate the AUC (Barve et al., 2011; Van Der Wal et al., 2009).

### 2.6 Model choice

We fit the presence and background training occurrence data to the predictor variables with a maximum entropy ecological niche model, Maxent 3.4.1 (Phillips et al., 2006; Phillips & Dudík, 2008), using package “dismo” (Hijmans et al., 2017). Maxent has been shown to consistently work well compared to profile and regression type models, and fits our research questions by allowing us to both hindcast and forecast our models (Elith & Graham, 2009; Elith et al., 2006). We fit the Maxent model with training data and predictor variables, estimated the amount of variance explained by each variable for the fitted Maxent model, and estimated the amount of explained variance lost by dropping out each variable in a jackknife analysis. The jackknife analysis quantified the relative contribution of each variable based on the performance of the overall model without the variable of interest and then compared it to a univariate model with only the variable of interest.

All models were run with both (1) default feature setting and regularization settings and (2) a regularization parameter set at 1 and no hinge feature, since Maxent models have been shown to be sensitive to the parameters of the algorithm (Hallgren et al., 2019; Phillips & Dudík, 2008; Radosavljevic & Anderson, 2014; Shcheglovitova & Anderson, 2013). We specifically chose the second scenario to ensure our models were not over‐fitted from using the hinge feature and a lower regularization parameter in scenario 1 (Radosavljevic & Anderson, 2014; Shcheglovitova & Anderson, 2013).

To evaluate our models, we folded the occurrence data by splitting it into separate testing and training sets. We folded the data in multiple ways, using both random folds and geographic folds. We evaluated the overall performance of the model by randomly folding the data into 80% training and 20% testing sets. However, splitting the data geographically informs how transferable the models are across time (Radosavljevic & Anderson, 2014; Roberts et al., 2017), so we developed four geographic folds by extracting 20% of the furthest out points along each of the cardinal directions as testing data and used the remaining 80% as training data.

One way we assessed model performance was using the area under the receiver operating characteristic curve (AUC). AUC is considered a reasonable and informative model if it is above 0.80 (Araujo et al., 2005; Swets, 1988); however, AUC is known to be biased (Fourcade et al., 2017; Peterson et al., 2008), so we also used the True Skill Statistic (TSS) at maximum sensitivity and specificity, which considers commission and omission errors and is independent of prevalence (Allouche et al., 2006). For TSS, values over 0.4 are considered reasonable (Araujo et al., 2005; Landis & Koch, 1977). Finally, to project the results of the models, we used an ensemble approach where we took the mean and variance of all the models for each time period and climate change scenario that met our AUC and TSS criteria.

### 2.7 Climate scenarios

We projected models meeting our evaluation criteria (AUC > 0.8 AND TSS > 0.4) onto two historic and four future climate scenarios represented as general circulation models (GCMs) from the Coupled Model Intercomparison Project 5 (CMIP5; Taylor et al., 2012), which include CCSM4, Community Climate System Model (Gent et al., 2011), and the MIROC‐ESM, Model for Interdisciplinary Research on Climate ‐ Earth System Model (Watanabe et al., 2011). We chose these GCMs, because they have projections for past climate as well as future climates. All of our past and future projections of the potential geographic distribution of *S. tergeminus* used the same sets of climate and environmental variables as the modern projections and were downloaded at their 2.5’ resolution. Bioclim and envirem variables are temporally dynamic (i.e., the variables change over a short time period, relative to the time periods used in the models), as they rely on measures or models of monthly climate and extraterrestrial solar radiation.

We used past climate models representing the mid‐Holocene and the Last Glacial Maximum. The mid‐Holocene lasted 7000 to 5000 years ago and during this time the summers were warmer and winters colder than modern (Bartlein et al., 2011; Steig, 1999). The Last Glacial Maximum occurred before 20,000 years ago when ice sheets were at their maximum extent (Clark et al., 2009). For each future climate scenario, we chose to project models onto GCMs derived from two representative concentration pathways (RCP + 2.6 and + 8.5 W/m^2^) for two time periods (2050 and 2070). The first scenario, RCP + 2.6 W/m^2^, assumes that global greenhouse gas emissions are presently at their peak (between 2010 and 2020) and will substantially decline after, which is an optimistic, yet unlikely, scenario (Meinshausen et al., 2011). The second scenario, RCP +8.5 W/m^2^, assumes that greenhouse gas emissions will continue to increase until 2100, which is a more realistic, but dire, scenario (Meinshausen et al., 2011). Both scenarios were averaged for two 20‐year periods: 2041–2060 (2050s) and 2061–2080 (2070s).

Finally, we calculated the mean and variance of the top selected Maxent model predictions to display the modeling results. We also calculated anomaly plots between the MIROC LGM and Current projections by subtracting the LGM raster from the current raster to highlight the differences between the two. We calculated the current and MIROC 2070 8.5 W/m^2^ similarly by subtracting the future raster from the current raster.

## 3 RESULTS

### 3.1 Model evaluation

**FIGURE 2:**
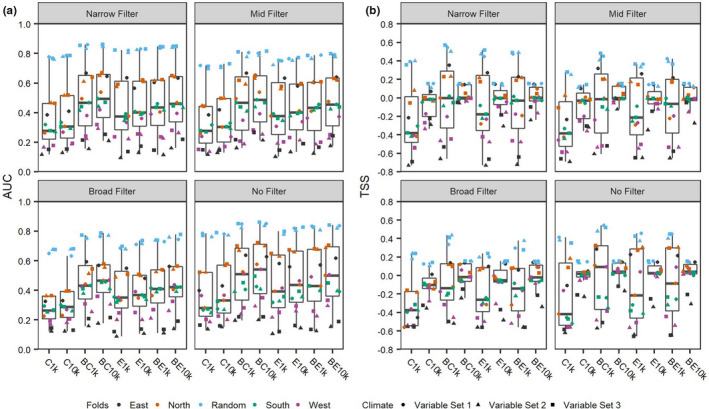
Model evaluation statistics for the full model set separated by different size filters (narrow filter, 0.15 bin; mid filter, 0.30 bin; broad filter 0.75 bin; and no filter). Background extent and number of background points are on the x‐axis. Climate variable sets and model testing folds are represented by different symbols and colors indicated in the legend. (a) Area under the receiver operating curve (AUC) and (b) True Skill Statistic (maximizing Sensitivity and Specificity) (TSS). Background extent abbreviations as follows: C1k, 100 km radius background with 1000 background points; C10k, 100 km radius background with 10,000 background points; BC1k, 200 km radius background, 1000 background points; BC10k, 200 km radius background, 10,000 background points; E1k, Minimum convex polygon (MCP) extent, 1000 points; E10k, MCP extent, 10,000 background points; BE1k, buffered MCP extent, 1000 points; BE10k, buffered MCP extent, 10,000 points. Climate variable sets from Table 1

Model performances varied greatly among the 480 Maxent models that we evaluated, depending on the particular combination of five key decisions made during the modeling process. In order of the contribution of decision type to variation in model performance, the decisions were (1) choice of random versus geographic folds for training and testing datasets, (2) number of background points, (3) size and shape of background polygon, (4) bin size of environmental filters, and (5) predictor variable selection (Figure 2).

Variation in model performance was mostly explained by choice of random versus geographic folds for training and testing datasets. Models with random folds had the highest evaluation statistics, by far. Models folded geographically by north and east were a distant next best and models folded geographically by south and west had very low evaluation statistics (Figure 2). The choice of the number of background points influenced TSS more than AUC. Background points of 10,000, rather than 1000, always resulted in a narrower distribution of TSS across models with varying folds, size and shape of background polygon, bin size of environmental filters, and predictor variable sets. Otherwise, both evaluation statistics, AUC and TSS, had similar results among the models (i.e., models that performed well evaluated by AUC generally performed well when evaluated by TSS).

Size and shape of background polygons, from which 1000 or 10,000 background points were drawn, produced variation in model performance. Larger background polygons consistently had higher evaluation statistics, regardless of the background polygon shape (either circular or buffered minimum convex polygon). For the smaller background polygons, shape mattered more than for the larger background polygons. Small circular shapes consistently had lower evaluation statistics than minimum convex polygons that were not buffered, but larger circular shapes had slightly better or equivalent evaluation statistics. Models with varying bin size of environmental filters do not seem to produce very different evaluation statistics, but models with no filter compared to models with some filters were consistently higher in their evaluation statistics. Differences in model performance due to predictor variable selection appear to have the smallest influence on the variation of the evaluation statistics.

We retained 16 of the 480 Maxent models (hereafter “top models”) that adequately discriminated between the test presence and background data (i.e., AUC > 0.8 & TSS > 0.4; Figure 2). The selected 16 models included all three variable sets, three of the four backgrounds, and only the random testing folds (Figure 2). None of the models that included the smaller 100 km circle buffers met the evaluation criteria and were not included in the top models.

### 3.2 Importance of climate and environmental variables

**FIGURE 3:**
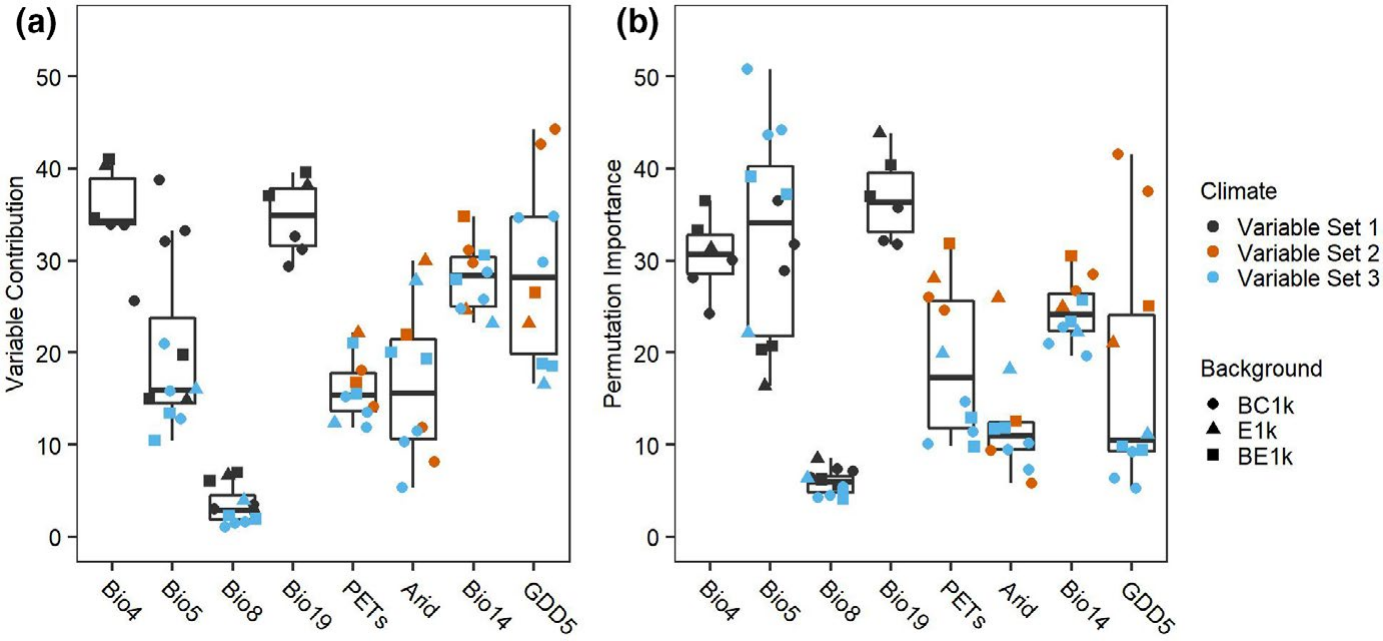
(a) Variable contribution and (b) permutation importance for each of the variables in the selected 16 distribution models for *Sistrurus tergeminus*, by climate variable set and background extent and points. Bioclim climate codes are: Bio4, temperature seasonality, Bio5, max temperature of the warmest month, Bio8, mean temperature of the wettest quarter, Bio14, precipitation of the driest month, and Bio19, precipitation of the coldest quarter. Envirem climate codes are: Arid, Thornthwaite aridity index, GDD5, growing degree days (5°C), PETs, potential evapotranspiration seasonality. Background extent abbreviations are: BC1k, 200 km radius background, 1000 background points; E1k, Minimum convex polygon (MCP) extent, 1000 points; BE1k, buffered MCP extent, 1000 points

For the top 16 Maxent models, there was variation in variable contributions and permutation importance of climate and environmental variables, although there was little variation due to the choice of background shape (Figure 3). Temperature seasonality and precipitation of the coldest quarter consistently contributed the most to the models, accounting for a mean 34.9% and 34.7% of the total variable contribution and a mean permutation importance of 30.6% and 36.8%, respectively, calculated from a jackknife procedure (Figure 3). Thornthwaite aridity index, growing degree days (5℃), potential evapotranspiration seasonality also had fairly high variable contribution, averaging between 16.1 and 29.0% (Figure 3). However, the permutation importance for these variables was wide ranging, with higher permutation importance for models with Variable Set 2, and lower importance for models with Variable Set 3 (Figure 3; Figure S1.1). Mean temperature of the wettest quarter contributed the least to the models and had a consistently low permutation importance.

The response plots, plots of the likelihood of occurrence along the gradient of each climate and environmental variable, showed that the highest probability of occurrence for *S. tergeminus* was for geographic localities that have between approximately 25 and 140 mm of precipitation during the coldest three months of the year, and a temperature seasonality ranging between 7 and 10°C, maximum temperatures between approximately 31 and 37°C during the hottest month of the year (Figure S1.2).

### 3.3 Model projections

**FIGURE 4:**
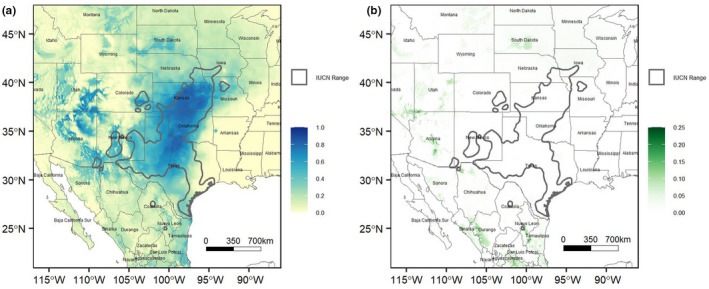
Current likelihood of occurrence projection for *Sistrurus tergeminus* estimated from the (a) mean and (b) variance of the selected 16 Maxent models

The averaged top models projected into geographic space are very similar to the range of *S. tergeminus*, capturing the high likelihood of occurrence across the core range of Texas, Oklahoma, and Kansas (Figure 4a). It also captures the locations of populations in southeastern Arizona, New Mexico, and southeastern Colorado. However, our model predicts high likelihood of occurrence outside the current range of *S. tergeminus*, especially in mid and northern Arizona, Utah, and Nevada. Variation across these models indicates consistent support (low variance) for high likelihoods of occurrence in the core of *S. tergeminus*’ range but much more variable support (high variance) for the high likelihoods of occurrence predicted for northern Arizona, Utah, and Nevada (Figure 4b).

**FIGURE 5:**
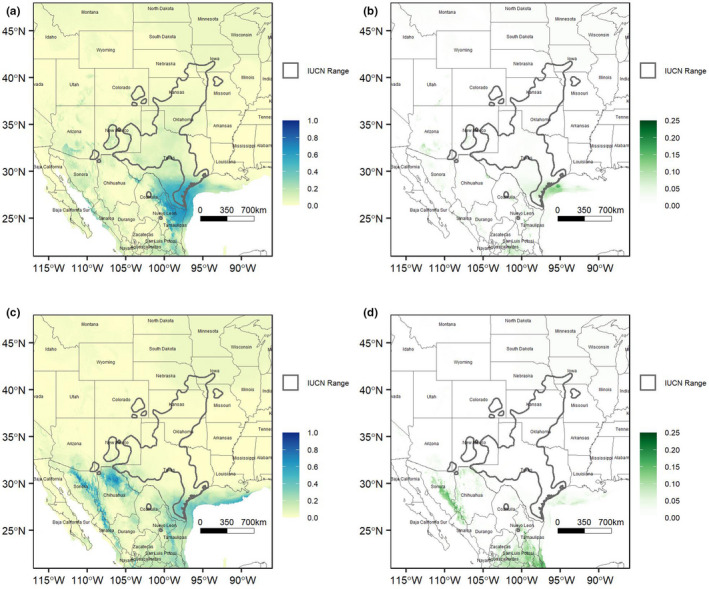
Likelihood of occurrence of *Sistrurus tergeminus* hindcast to the last glacial maximum, using the selected 16 Maxent models. (a) Mean and (b) variance for the CCSM4 global circulation model (GCM). (c) Mean and (d) variance for the MIROC‐ESM GCM

Hindcasting the top models on mid‐Holocene GCMs 7000 to 5000 years ago shows a similar distribution for *S. tergeminus* compared to the modern distribution (Figure S1.3). While there are some areas projected to have a high likelihood of occurrence to the northeast of the current range (i.e., South Dakota, Nebraska, Iowa, Missouri, Illinois), those areas have a relatively high variance (Figure S1.3B,D) showing those projected areas were variable among the individual model predictions. However, projecting the same models on Last Glacial Maximum GCMs more than 20,000 years ago shows dramatically decreased areas of suitable climates and environments in most of the US, but increased areas of suitable climates and environments in South Texas (Figure 5a,b CCSM4 models) and Mexico (Figure 5c,d MIROC‐ESM models).

**FIGURE 6:**
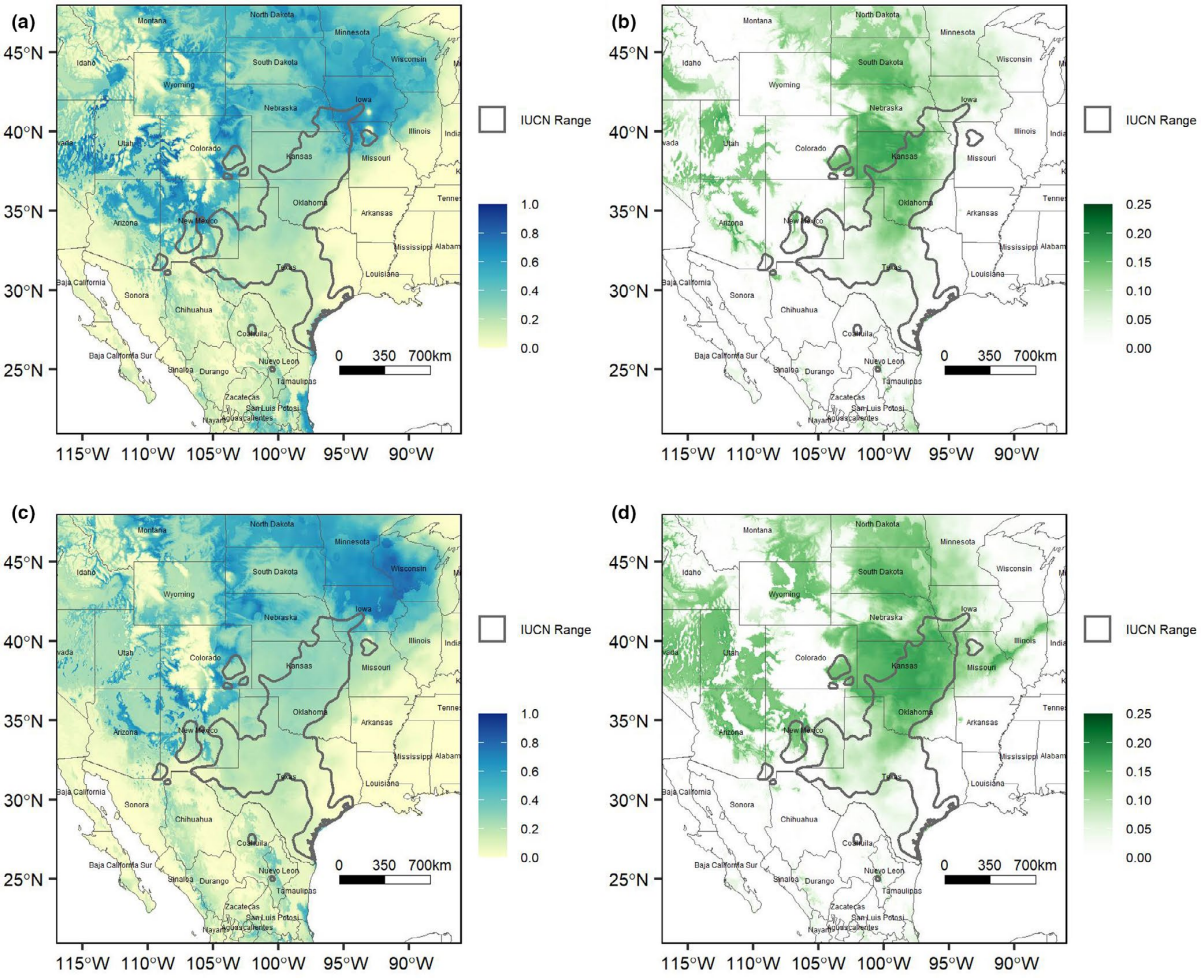
Likelihood of occurrence of *Sistrurus tergeminus* forecast under a 2070 8.5 W/m^2^ warming scenario, estimated from the selected 16 Maxent model. (a) Mean and (b) variance for the CCSM4 global circulation model (GCM). (c) Mean and (d) variance for the MIROC‐ESM GCM

**FIGURE 7:**
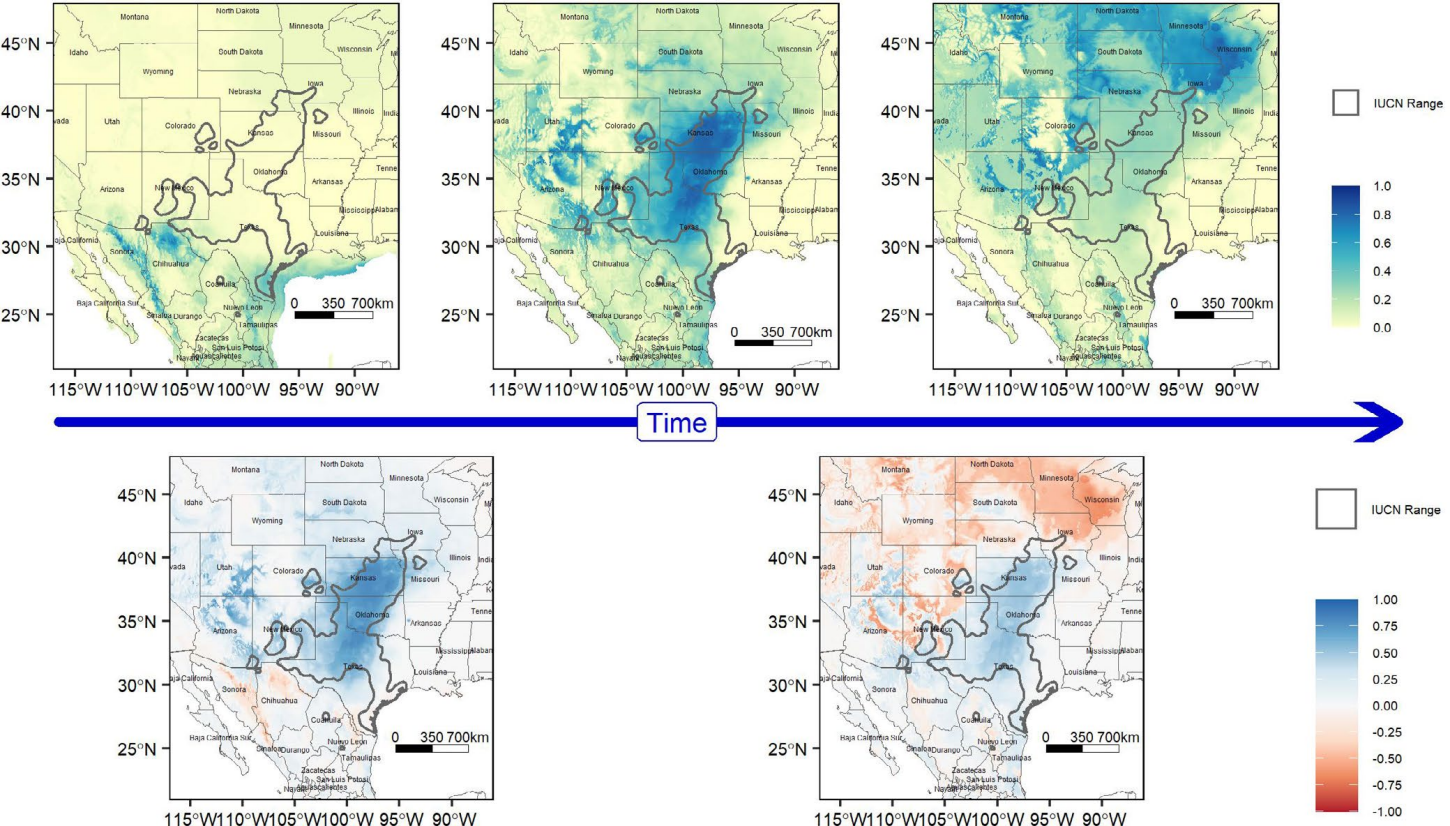
Changes in the mean likelihood of occurrence of *Sistrurus tergeminus* over time, estimated from the selected 16 Maxent models. Top row, left to right: last glacial maximum model (LGM), current predictions, and the 8.5 W/m^2^ in 2070. Bottom row, left to right: Anomaly map showing difference between LGM and current predictions (positive values represent higher likelihood of occurrence currently, with negative values historic) and anomaly map between current predictions and 8.5 W/m^2^ in 2070 (positive values current, negative values future). Historic and future projections based off of the MIROC‐ESM global circulation model

Top models projected onto future climate scenarios show geographic shifts in potential geographic distribution across the entire range of *S. tergeminus* and great variability between RCPs for 2050 and 2070 (Figures S1.4–1.6). As expected, there was greater variation between RCPs than between GCMs, which indicates that the RCP choice was relatively more important to consider than GCM when assessing potential shifts in suitable habitat. For the 2.6 W/m^2^ RCP, there is a relatively small shift northward in the range of *S. tergeminus*, shifting the center of its range to Kansas‐Nebraska‐Iowa‐Missouri. This shift is much larger in the 8.5 W/m^2^ RCP, where the core of the range shifts from Texas‐Oklahoma‐Kansas to Missouri‐Nebraska‐Iowa‐Eastern Colorado in 2050 and up further to North Dakota‐Minnesota‐Iowa‐Wyoming for 2070 projections (Figures 6 and 7). There are small shifts in suitable habitat between the GCMs, with more of an east‐west split occurring across Kansas and Nebraska and a few southern coastal populations remaining climatically viable (e.g., Padre Island and Matagorda Island populations in Texas) for the CCSM4 models.

## 4 DISCUSSION

### 4.1 Model performance and projections

The averaged top models were able to capture the current range of *S. tergeminus* quite well, but there was a wide range of variation in model performance from various choices made throughout the modeling process. These choices are often unexplored in detail and evaluating all combinations of these five key choices allowed us to evaluate which choices had the most influence on model performance for *S. tergeminus*. We found that the choice of random versus geographic folds for training and testing datasets had the largest influence on the variation of model performance. The number of background points, size and shape of background polygons, and use of environmental filters contributed some variation in model performance. Predictor variable sets contributed the least to model performance. For the top models, a few key variables like precipitation of the coldest quarter (Bio19), temperature seasonality (Bio4), and max temperature of the warmest month (Bio5) helped describe the distribution across the range.

Our models showed some weak support (i.e., high variance) for an available climate and environmental space in Utah, Northern Arizona, and Nevada (Figure 4); however, this species has never been documented west of the Rocky Mountains or beyond the southeast corner of Arizona. While these areas may be climatically similar to other parts of the range of *S. tergeminus*, there has apparently been a barrier to dispersal in those areas for this grassland species, most likely high mountain ranges or the grassland biome is not present in those projected areas of suitable climate and environment. A third possibility is that populations were able to disperse and live there, but have been extirpated from that part of the geographic distribution of the species.

Only our models using random background points ranked as top models due to considerable variation across geographic folds. The geographic folds showed variation in which climate variables had higher variable contributions and permutation importance (Figure S1.1), indicating that the species was not responding to the same climate cues consistently across its large range. This suggests that subsets of our model results are not transferable in space, likely due to gradients in aridity and precipitation, as well as temperatures. For example, Thornthwaite's aridity index (Arid) and temperature seasonality (Bio4) had a higher permutation importance in the south and the west, while maximum temperature of the warmest month (Bio5) and growing degrees day (GDD5) had a higher permutation importance in the north and east. This geographic variation in variable importance most likely reflects the north to south shifts in precipitation systems and mean annual temperatures, as well as the rain shadow precipitation gradient extending east from the foothills of the Rocky Mountains through the Midwestern United States, that shaped the current structure and distribution of the North American grassland biome (Anderson, 2006; Roy et al., 2019). For a grassland reptile, aridity and precipitation, as drivers of drought, are more likely to play a role in limiting the distribution of the species in the southwest (Holycross, 2020; Prugh et al., 2018). Comparatively, in the northern parts of its range, temperature is likely to be a limiting factor in the occupied geographic distribution of *S. tergeminus*, as it is for its sister species, *S. catenatus*, which is found in the northern United States and southern Canada (Harvey & Weatherhead, 2010).

All decisions within the modeling process were made following a rubric for model development to help assess the reliability of various aspects of the model for conservation decision‐making (Sofaer et al., 2019). We did not develop a full species distribution model, which would have incorporated another process to trim the modeled potential geographic distribution to a model of the actual distribution of the species. Instead, we choose to focus on the availability of suitable climate and environment through time and the evaluation of multiple decisions during the modeling process. Overall, we assessed our model to be acceptable in the four components identified by Sofaer et al. (i.e., quantity and quality of species data, attributes of environmental predictors, attributes of the modeling process, and attributes of model products) based on our consideration of the modeling decisions and criteria described therein (Table S1.1). Future work incorporating dispersal dynamics and population demographics would contribute to further understanding of the threats to the survival of this species.

### 4.2 Paleobiogeographic scenario

During the LGM, the overall availability and seasonal distribution of moisture and temperatures produced a Great Plains dominated by park‐like, open‐canopy coniferous woodlands with areas of boreal spruce forest stretching through the Central Plains to the Appalachian Mountains (Hafsten, 1961; Wendorf, 1961; Wright et al., 1987). To the southwest, pollen and plant remains from woodrat (*Neotoma* spp.) middens indicate Chihuahuan Desert grasslands experienced equable climates during the LGM (Holmgren et al., 2007; Van Devender & Spaulding, 1979; Wells, 1966), which lowered elevational and southern range limits of pinyon‐juniper‐oak woodlands without extirpating endemic desert scrub succulents and subtropical plants, resulting in vegetation assemblages that have no modern analogs (Van Devender, 1990). In general, the climate of southwestern North America during the LGM may have been as much as 5°C cooler than today, with greater winter precipitation (Asmerom et al., 2010; Menking et al., 2004). Like many other grassland vertebrate taxa from this region (e.g., Graham, 2005; Williford et al., 2013; Zink et al., 2001), we found that there was reduced availability of suitable climate and environment space during the LGM for *S. tergeminus* and that *S. tergeminus* would have had to track suitable climate southward into northern Mexico, and the present‐day borderlands of southern Texas and eastern Mexico to survive (Figure 5).

As this species tends to avoid woodlands and areas with dense shrubs (Mackessy, 2005), its historical distribution probably fluctuated repeatedly as periodic climatic changes resulted in shifting elevation and southern range limits of pinyon‐juniper‐oak woodlands. Open grasslands may not have been widely distributed in southwestern North America until the onset of warmer, drier conditions in the region at the end of the LGM (Holmgren et al., 2007). Indeed, after the LGM, *S. tergeminus* most likely expanded quickly to approximately its current range through the mid‐Holocene (Figure 7) tracking the spread of grasslands and desert thornscrub to their pre‐ and post‐glacial distributions (Hafsten, 1961; Hoyt, 2000; Wendorf, 1961; Wright et al., 1987).

Pleistocene glacial cycles and geographic barriers have had different effects on arid‐ and desert‐adapted vertebrate taxa of southwestern North America, where some species exhibit strong phylogeographic structure due to isolation in multiple refugia during cooler, wetter glacials (e.g., Andersen & Light, 2012; Castoe et al., 2007; Douglas et al., 2006; McGuire et al., 2007; Neiswenter & Riddle, 2010, 2011; Pyron & Burbrink, 2010; Riddle & Hafner, 2006; Riddle et al., 2000; Zamudio et al., 1997) and other species show evidence of recent expansion from a single refugium (Douglas et al., 2006; Riddle & Hafner, 2006; Williford et al., 2013; Zink et al., 2001; Zink, 2002). While these glacial cycles date back millions of years, previous phylogenetic research indicates a recent origin for many lineages in the genus *Sistrurus*, including *S. tergeminus* (Kubatko et al., 2011), beginning in the late Pleistocene rather than earlier geological eras, which apparently drove diversification of other North American snakes (i.e., late Miocene and Pliocene; see Bryson et al., 2007; Burbrink et al., 2000; Castoe et al., 2007; Douglas et al., 2006; Fontanella et al., 2008; Pook et al., 2000). Recent genetic analyses showed that *S. tergeminus* exhibits a star‐like haplotype network (Ryberg et al., 2015) that is indicative of a recent, rapid demographic expansion from a single compact refugium (Slatkin & Hudson, 1991). The paleogeographic reconstruction detailed above offers one explanation of these genetic results, where *S. tergeminus* colonized much of its current range relatively recently, coincident with the expansion of grasslands and desert thornscrub at the end of the LGM (Hoyt, 2000; Metcalfe, 2006; Wright et al., 1987).

Given the poor sampling across the fossil record for this species (Parmley & Hunter, 2010), it is difficult to use such data to pinpoint exactly where a compact Pleistocene refugium may have existed. That said, the distribution of haplotypes can provide qualitative insights into the location of Pleistocene refugia (Provan & Bennett, 2008; Waltari et al., 2007; Wilson & Pitts, 2012). The highest haplotype diversity occurred within the Southern Plains of Texas and eastern New Mexico. This high diversity may indicate that *S. tergeminus* was restricted to this region during part of the Pleistocene and that the rest of the *S. tergeminus* range resulted from recent colonization during the Holocene. Lower genetic diversity is expected at the periphery of the expansion due to the loss of haplotypes through founder events and local bottlenecks (Austerlitz et al., 1997). In addition, modern plant communities were present in Texas by the Late Holocene (~6000 years ago), and similar vegetation may have been present in southern Texas as early as the Late Pleistocene (Bryant & Holloway, 1985). Finally, *S. tergeminus* is not presently known from northern Mexico (Sonora or Chihuahua), but still occupies much of the southernmost tip of the Great Plains, which extends from southern Texas into eastern Mexico (Coahuila and Nuevo Leon). Thus, the most logical location for a Pleistocene refuge for *S. tergeminus* would be the latter. If additional Pleistocene refuges did exist in northern Mexico for example, then the lack of genetic structure observed in recent studies would suggest that *S. tergeminus* populations using those refuges simply did not persist.

### 4.3 Future implications

Identifying this Pleistocene refuge contributes to our understanding of current *S. tergeminus* population distribution and genetic structure as described above, but given that glaciation is not projected as a direction of future climate change in the region, this Pleistocene refuge has a very low likelihood of persisting in the near future. Instead, for future climates, we see an extensive predicted shift of the *S. tergeminus* distribution northward, especially under the 8.5 W/m^2^ RCP (Figure 7). This distributional shift includes both an expansion of the species’ range north and a retraction from the southern limits of the species’ range in eastern Mexico, and southern Texas, New Mexico, and Arizona. Tracking available climate for this grassland species may present distinct conservation issues along northern and southern range boundaries (Cagle, 2008; Gedir et al., 2015).

Currently, the northern edge of the range of *S. tergeminus* lies in the southern part of Nebraska. With a potential shift north into South and North Dakota, and east into Iowa, Wisconsin, and Minnesota, this species could be tracking suitable climate into areas with considerable agriculture. *Sistrurus tergeminus* and many other grassland snake species are known to be absent from agricultural lands, preferring open grasslands across their range (Cagle, 2008; Mackessy, 2005; Patten et al., 2016). Unfortunately, grasslands in Iowa, eastern Minnesota, western Nebraska, and South Dakota are highly fragmented from agricultural development (Samson et al., 2004), and grassland connectivity across suitable climate is broken within the current and future range of *S. tergeminus* (McGuire et al., 2016; Figures S1.7). Assisted migration through the creation of climate corridors or physical translocation of *S. tergeminus* individuals to pockets of suitable climate and environment may be required along the species’ shifting northern boundary to balance population losses from the retracting southern range boundary over the next century. There is greater variation between RCPs than between GCMs, which indicates that the RCP choice is relatively more important to consider than GCM when assessing potential shifts in suitable habitat.

Along the retracting southern boundary, so‐called “rear” populations in eastern Mexico, southern Texas, New Mexico, and in southeast Arizona may disappear faster than expected due to continued habitat degradation from shrub invasion and desertification (Hampe & Petit, 2005). Indeed, several historic populations in Arizona have apparently been extirpated already (Holycross, 2020). Additionally, while the complex topography of the basin and range province does not appear to have played a role in creating population and genetic structure during the LGM, it may yet shape the distribution of *S. tergeminus* populations under future climate change by creating physical or ecogeographic barriers to northward expansion or inter‐basin dispersal. Far to the east of the basin and range province in coastal Texas, barrier islands are predicted to maintain suitable climate and could serve as a refuge for *S. tergeminus* populations in the future (Figure 6). However, adjacent mainland areas are not predicted to be climate refuges, and the potential loss of northward moving source populations could reduce immigration and genetic connectivity and thus further fragment the *S. tergeminus* distribution. As opposed to assisting migration in the north, future *S. tergeminus* conservation efforts along the southern range boundary should focus on restoring and managing quality habitats in predicted climate refugia.

However, all these predictions are limited based on limitations in modeling shifts in species distributions using maximum entropy to model the ecological niche and potential geographic distribution. Beyond some of the modeling choices described above, limitations include effects from abiotic variables such as non‐analog climates (climate conditions that do not currently exist), limited ability to predict land‐use change (here we compared current land use to future potential distributions), and potential scale mismatch (Austin & Van Niel, 2011; Fitzpatrick & Hargrove, 2009; Seo et al., 2009; Sinclair et al., 2010). There are also limitations in knowledge of a species biology, including incomplete sampling of the niche space, potential interspecific interactions, limited understanding of species mobility and capacity to emigrate, and the potential for evolution and adaptation (Heikkinen et al., 2007; Massot et al., 2008; Sinclair et al., 2010).

## 5 CONCLUSIONS

The lack of genetic variation range‐wide indicates that *S. tergeminus* was forced into a single Pleistocene refugium, which, according to model projections in this study, was most likely restricted to eastern Mexico and southern Texas. The apparent expansion of suitable climate and environment from the mid‐Holocene to modern day resulted in the current distribution of *S. tergeminus* populations, which follow an isolation by distance model of genetic structure (Bylsma et al., 2021). These data support the theory that *S. tergeminus* was fully capable of tracking changes in their distribution in response to past climate change, rather than evolving absolute climate tolerances to persist. Under future climate scenarios, models predicted that suitable *S. tergeminus* climate will expand north, but also retract from the south. Ideally, the potential loss of southern *S. tergeminus* populations will be compensated for by the predicted northern expansion of populations. However, the success of such a scenario will undoubtedly rely on the kind of human interventions described above, as the capacity and ability of *S. tergeminus* populations to deliver such an expansion northward through modern landscapes is limited by habitat loss and fragmentation from agriculture. Indeed, if the distributional response of *S. tergeminus* to climate change is constrained by natural and human barriers anywhere within its extensive geographic range, then the rate of climate change may outpace the species’ capacity to adjust in those areas, leading to rapid localized changes in the size and distribution of *S. tergeminus* populations. As such, *S. tergeminus* could be highly vulnerable to future changes in climate in specific regions throughout its current distribution.

## CONFLICT OF INTEREST

The authors have no conflicts of interest.

## AUTHOR CONTRIBUTIONS

**Danielle K. Walkup:** Data curation (equal); Formal analysis (equal); Funding acquisition (equal); Methodology (equal); Visualization (equal); Writing – original draft (equal); Writing – review & editing (equal). **Anna Michelle Lawing:** Conceptualization (equal); Formal analysis (equal); Funding acquisition (equal); Methodology (equal); Supervision (equal); Visualization (equal); Writing – original draft (equal); Writing – review & editing (equal). **Toby J. Hibbitts:** Conceptualization (equal); Data curation (equal); Funding acquisition (equal); Supervision (equal); Validation (equal); Writing – review & editing (equal). **Wade A. Ryberg:** Conceptualization (equal); Funding acquisition (equal); Investigation (equal); Project administration (equal); Supervision (equal); Writing – original draft (equal); Writing – review & editing (equal).

## Supporting information

Supplementary Material

## Data Availability

All scripts for data analyses and species distribution data have been uploaded to Dryad (https://doi.org/10.5061/dryad.m37pvmd3t).
