# Supplementary material for: Biogeographic consequences of shifting climate for the western massasauga (Sistrurus tergeminus)
Source: Ecol Evol. 2022 Feb 10;12(2):e8599. doi: 10.1002/ece3.8599 (PMC8831096; doi:10.1002/ece3.8599)
Supplement: Supplementary file 1 — Supplementary Material [file ECE3-12-e8599-s001.docx]

# **Supplementary Information**

Table of Contents

[**Tables** 3](#_Toc89065193)

[Table S1.1. Model evaluation based on Tables 1a-d in Sofaer et al. (2019). Assessment is the authors’ assessments (Interpret with Caution, Acceptable, or Ideal) based on the stated criteria, supported with the Reasoning and Citations. 3](#_Toc89065194)

[**Figures** 6](#_Toc89065195)

[Figure S1.1. (a) Variable Contribution and (b) Permutation Importance for each of the variables in all 480 distribution models for *Sistrurus tergeminus*, by climate variable set and background extent and points. Bioclim climate codes are: Bio4 = temperature seasonality, Bio5 = max temperature of the warmest month, Bio8 = mean temperature of the wettest quarter, Bio14 = precipitation of the driest month, and Bio19 = precipitation of the coldest quarter. Envirem climate codes are: Arid = Thornthwaite aridity index, GDD5 = growing degree days (5°C), PETs = potential evapotranspiration seasonality. Background extent abbreviations as follows: BC1k = 200 km radius background, 1,000 background points; E1k = Minimum convex polygon (MCP) extent, 1,000 points; BE1k = buffered MCP extent, 1,000 points. 6](#_Toc89065196)

[Figure S1.2. Variable response curves for each of the climate predictor variables included in the selected 16 models. Variable Set 1 = Bio4 - temperature seasonality, Bio5 - max temperature of the warmest month, Bio8 - mean temperature of the wettest quarter, and Bio19 - precipitation of the coldest quarter; Variable Set 2 = Bio14 - precipitation of the driest month, Arid - Thornthwaite aridity index, GDD5 - growing degree days (5°C), and PETs - potential evapotranspiration seasonality; Variable Set 3 = Bio5, Bio8, Bio14, Arid, GDD5, and PETs. 7](#_Toc89065197)

[Figure S1.3. Likelihood of occurrence of *Sistrurus tergeminus* hindcast to the mid-Holocene, using the selected 16 Maxent models. (a) Mean and (b) Variance for the CCSM4 global circulation model (GCM). (c) Mean and (d) Variance for the MIROC-ESM GCM. 8](#_Toc89065198)

[Figure S1.4. Likelihood of occurrence of *Sistrurus* *tergeminus* forecast under a 2050 2.6 W/m2 warming scenario, estimated from the selected 16 Maxent models. (a) Mean and (b) Variance for the CCSM4 global circulation model (GCM). (c) Mean and (d) Variance for the MIROC-ESM GCM. 9](#_Toc89065199)

[Figure S1.5. Likelihood of occurrence of *Sistrurus* *tergeminus* forecast under a 2050 8.5 W/m2 warming scenario, estimated from the selected 16 Maxent models. (a) Mean and (b) Variance for the CCSM4 global circulation model (GCM). (c) Mean and (d) Variance for the MIROC-ESM GCM. 10](#_Toc89065200)

[Figure S1.6. Likelihood of occurrence of *Sistrurus* *tergeminus* forecast under a 2070 2.6 W/m2 warming scenario, estimated from the selected 16 Maxent models. (a) Mean and (b) Variance for the CCSM4 global circulation model (GCM). (c) Mean and (d) Variance for the MIROC-ESM GCM. 11](#_Toc89065201)

[Figure S1.7. (a) Likelihood of occurrence over 50% in green plotted on the National Land Cover map (Homer, Fry, & Barnes, 2012) under the CCSM4 2070 8.5 W/m GCM. (b) Frequency of land cover classes in the green polygons for the potential shift in climate space into South Dakota, North Dakota, Nebraska, Montana, Wyoming, Iowa, Wisconsin, and Minnesota. This shows that approximately 50% of the space that presents as a suitable climate under the future scenarios may not be available as grassland habitat, as it is already cultivated crops or pasture/hay. 12](#_Toc89065202)

[**References** 13](#_Toc89065203)

# **Tables**

### Table S1.1. Model evaluation based on Tables 1a-d in Sofaer et al. (2019). Assessment is the authors’ assessments (Interpret with Caution, Acceptable, or Ideal) based on the stated criteria, supported with the Reasoning and Citations.

|  | Assessment | Reasoning | Citations |
| --- | --- | --- | --- |
| *Table 1a.* | | | |
| Presence Data | Acceptable | 1. Spatial error of points (< 1km) smaller than spatial grain of model (2.5’ or ~ 4.5 km).  2. All variations on historical names were clarified and all samples in the model are western massasauga (*Sistrurus tergeminus*).  3. Species experts (TJH) verified presences and removed any questionable outliers.  4. Presence points were filtered environmentally, with 4 presence data options used (unfiltered, small, medium, and large filters). | 4. Varela, Anderson, García-Valdés, & Fernández-González, 2014 |
| Absence or Background Data | Acceptable | Sensitivity analyses were used to evaluate effects of different background data sets: 8 different sets, with 4 background extents and 2 different numbers of points (1000, 10000). | Barbet-Massin, Jiguet, Albert, & Thuiller, 2012; Barve et al., 2011; Jarnevich et al., 2017; Phillips et al., 2009 |
| Evaluation Data | Acceptable | Evaluated using random and spatial 5-fold cross-validation. | Radosavljevic & Anderson, 2014; Roberts et al., 2017 |
| *Table 1b.* | | | |
| Ecological and predictive relevance | Acceptable | 1. Climate predictors chosen based on question and methodology  2. Climate variables are important for ectotherms and their habitat (grasslands) |  |
| Spatial and Temporal Alignment | Acceptable | 1. Predictors encompass the study area at a reasonable resolution  2. Predictors cover a subset of the time period (1960-1990) that the distribution records cover (1912 – 2019), with the majority of the records occurring from 1960 – 2019. |  |
| *Table 1c.* | | | |
| Algorithm choice | Acceptable | MaxEnt was chosen because it has been shown to consistently work well compared to profile and regression type models, and fits our research questions by allowing us to both hindcast and forecast our models | Elith & Graham, 2009; Elith et al., 2006 |
| Sensitivity | Acceptable | Only one algorithm was used (MaxEnt), but we tested the sensitivity of that model to the beta-regularization parameter, function choice (with and without hinge), and number of background points | Hallgren, Santana, Low-Choy, Zhao, & Mackey, 2019; Phillips et al., 2009; Radosavljevic & Anderson, 2014; Shcheglovitova & Anderson, 2013 |
| Statistical rigor | Acceptable | Assumptions recognized and considered | Phillips, Anderson, & Schapire, 2006; Table 1a-b |
| Performance | Acceptable | 1. Multiple metrics were evaluated (AUC and TSS), with only the models meeting the generally accepted levels retained.  2. Ecological plausibility evaluated by species experts | 1. Allouche, Tsoar, & Kadmon, 2006; Araujo, Pearson, Thuiller, & Erhard, 2005; Landis & Koch, 1977 |
| Model review | Caution | Review by co-author species expert (TJH). |  |
| Iterative | Ideal | 1. Updated based on performance assessment (changed from Ryberg et al., 2017).  2. Targeted field sampling performed in gaps, new records added (2018 – 2019) | 1. Ryberg, Lawing, & Hibbitts, 2017  2. Ryberg et al., 2020 |
| *Table 1d.* | | | |
| Mapped products | Ideal | 1. Continuous predictions mapped with description  2. Exploration of sensitivity  3. Mapping of uncertainties and extrapolation – Anomaly map |  |
| Interpretation and support products | Acceptable | 1. Every row in Sofaer et al. 2019 Table 1a-d has been addressed.  2. Variables are described in methods and results |  |
| Reproducibility | Acceptable | 1. Inputs saved and made available (except rare species locations), with permissions  2. Scripts archived on GitHub | https://git.io/JL831 |
|  |  |  |  |

# **Figures**


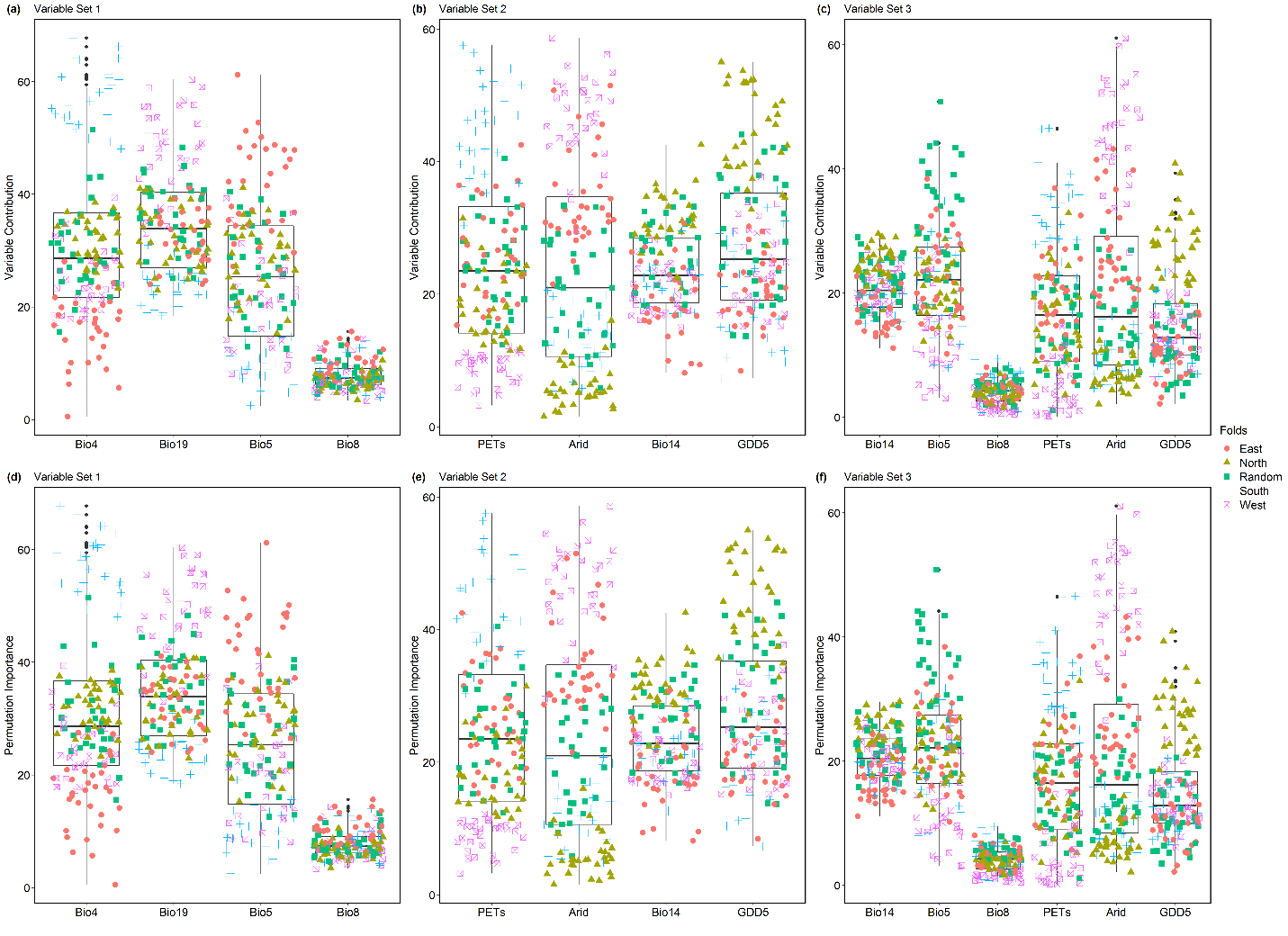


### Figure S1.1. (a) Variable Contribution and (b) Permutation Importance for each of the variables in all 480 distribution models for *Sistrurus tergeminus*, by climate variable set and background extent and points. Bioclim climate codes are: Bio4 = temperature seasonality, Bio5 = max temperature of the warmest month, Bio8 = mean temperature of the wettest quarter, Bio14 = precipitation of the driest month, and Bio19 = precipitation of the coldest quarter. Envirem climate codes are: Arid = Thornthwaite aridity index, GDD5 = growing degree days (5°C), PETs = potential evapotranspiration seasonality. Background extent abbreviations as follows: BC1k = 200 km radius background, 1,000 background points; E1k = Minimum convex polygon (MCP) extent, 1,000 points; BE1k = buffered MCP extent, 1,000 points.


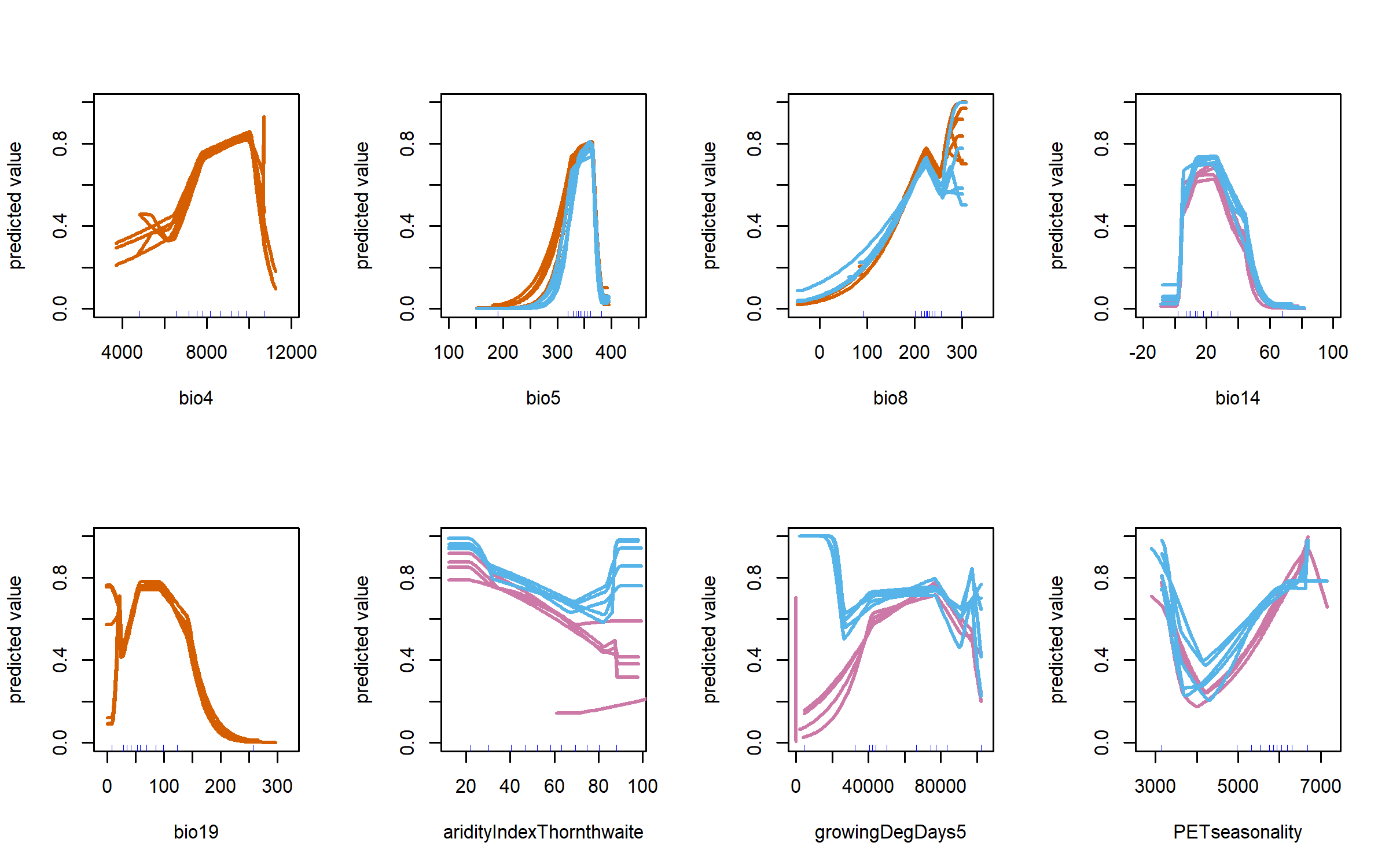


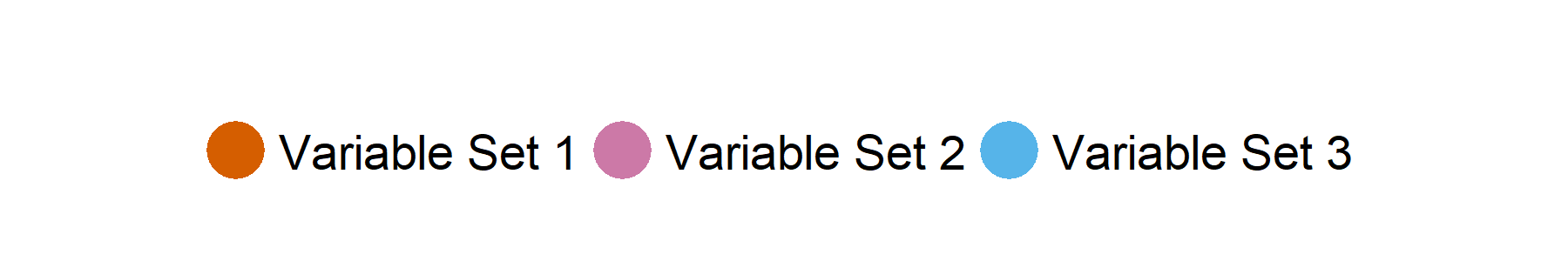


### Figure S1.2. Variable response curves for each of the climate predictor variables included in the selected 16 models. Variable Set 1 = Bio4 - temperature seasonality, Bio5 - max temperature of the warmest month, Bio8 - mean temperature of the wettest quarter, and Bio19 - precipitation of the coldest quarter; Variable Set 2 = Bio14 - precipitation of the driest month, Arid - Thornthwaite aridity index, GDD5 - growing degree days (5°C), and PETs - potential evapotranspiration seasonality; Variable Set 3 = Bio5, Bio8, Bio14, Arid, GDD5, and PETs.


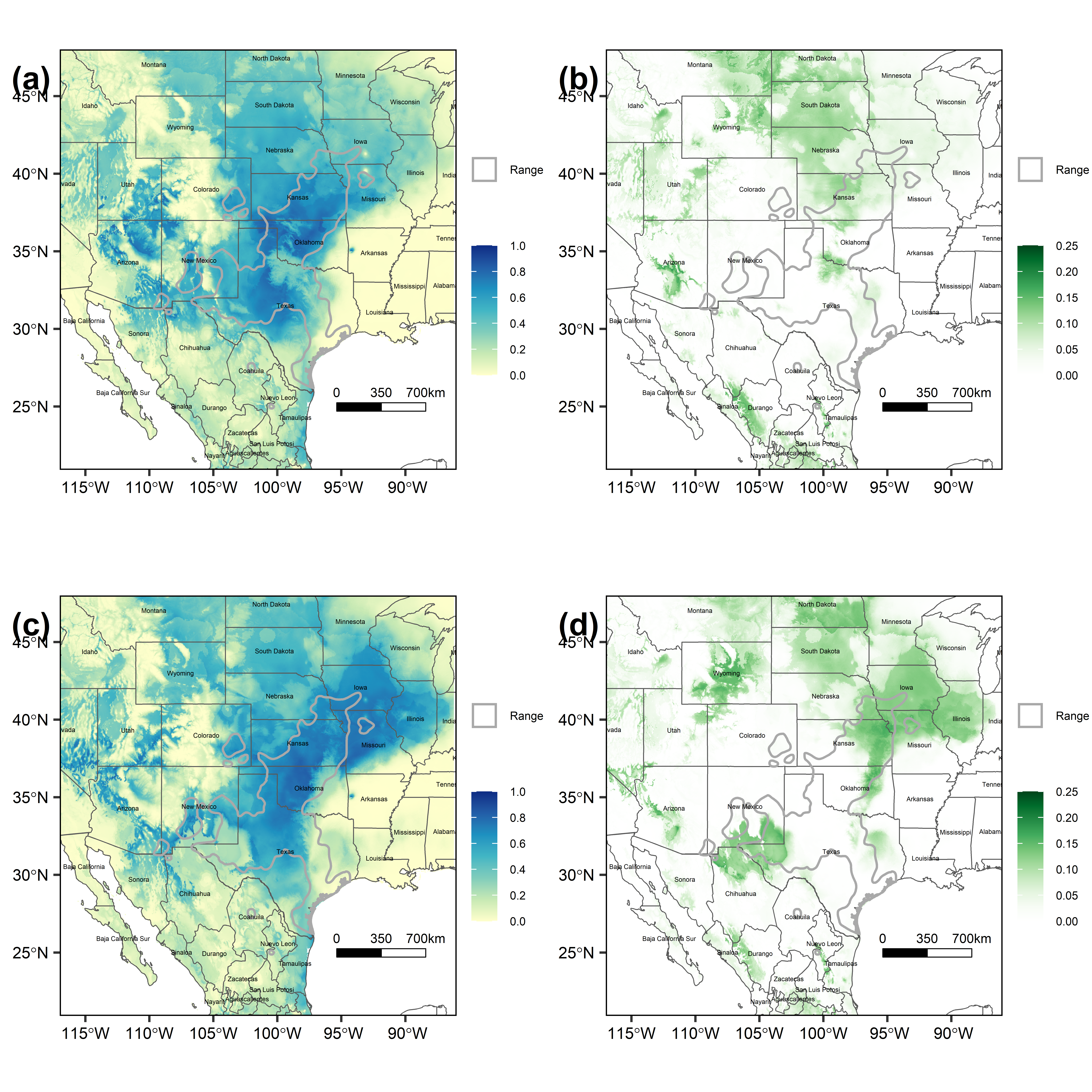


### Figure S1.3. Likelihood of occurrence of *Sistrurus tergeminus* hindcast to the mid-Holocene, using the selected 16 Maxent models. (a) Mean and (b) Variance for the CCSM4 global circulation model (GCM). (c) Mean and (d) Variance for the MIROC-ESM GCM.


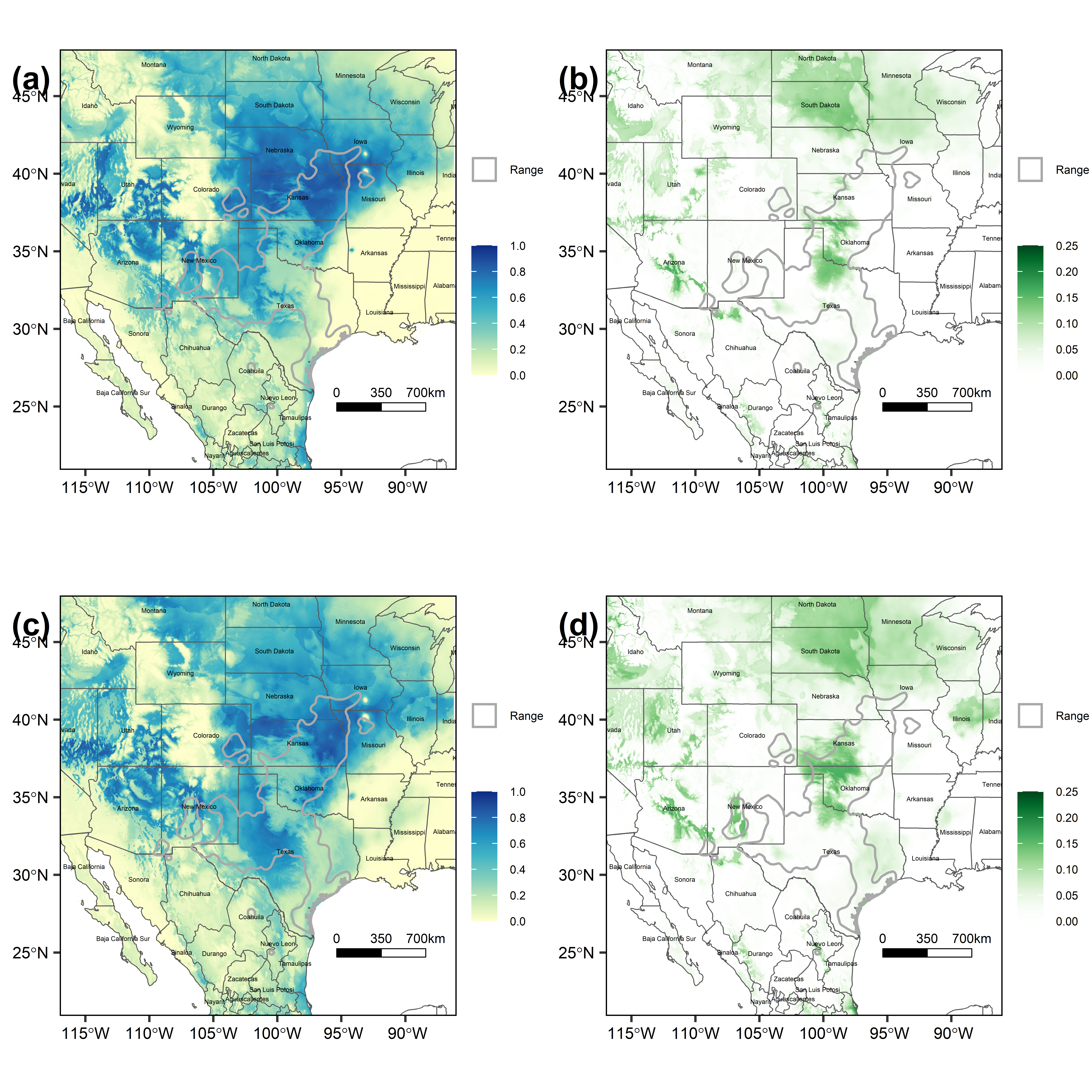


### Figure S1.4. Likelihood of occurrence of *Sistrurus* *tergeminus* forecast under a 2050 2.6 W/m2 warming scenario, estimated from the selected 16 Maxent models. (a) Mean and (b) Variance for the CCSM4 global circulation model (GCM). (c) Mean and (d) Variance for the MIROC-ESM GCM.


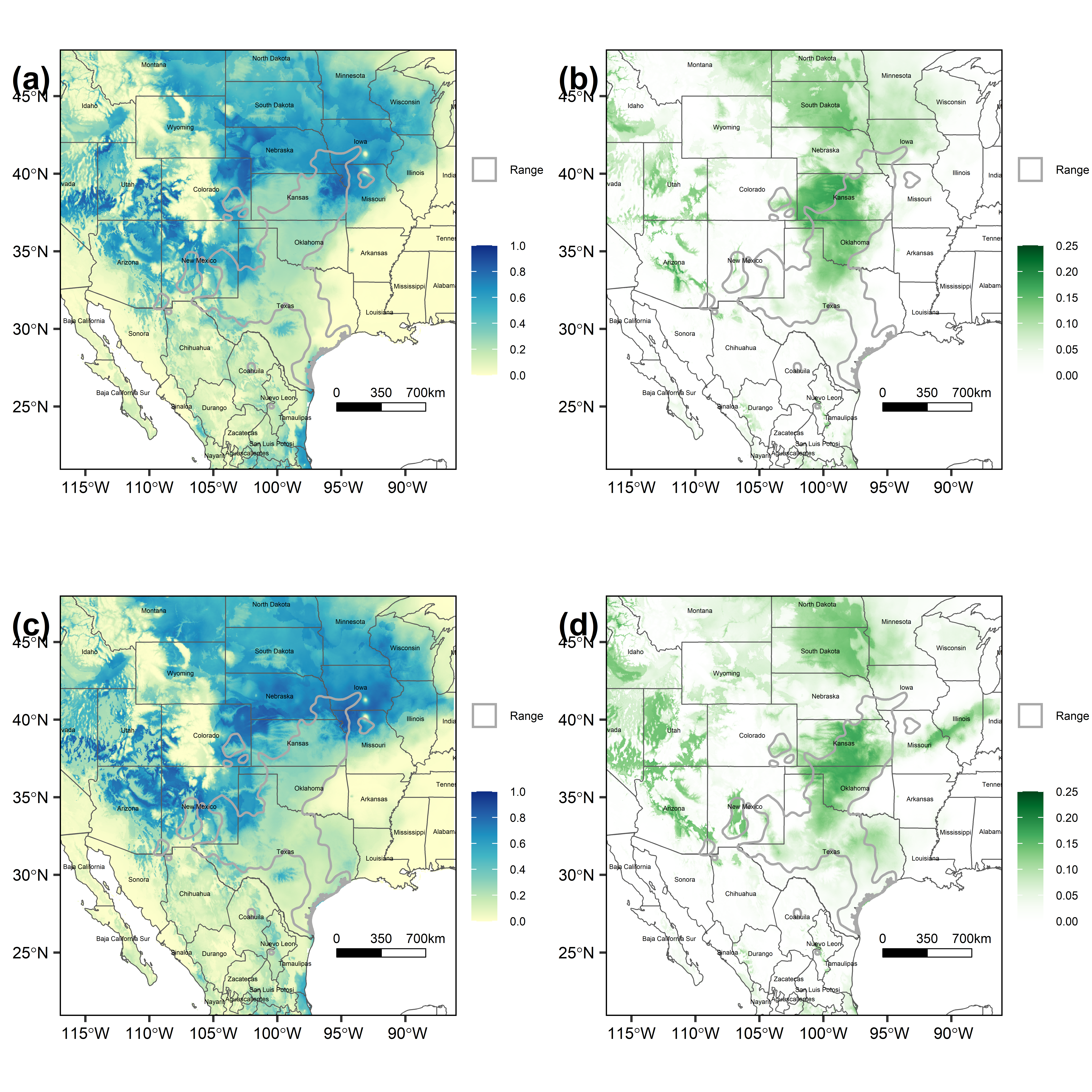


### Figure S1.5. Likelihood of occurrence of *Sistrurus* *tergeminus* forecast under a 2050 8.5 W/m2 warming scenario, estimated from the selected 16 Maxent models. (a) Mean and (b) Variance for the CCSM4 global circulation model (GCM). (c) Mean and (d) Variance for the MIROC-ESM GCM.


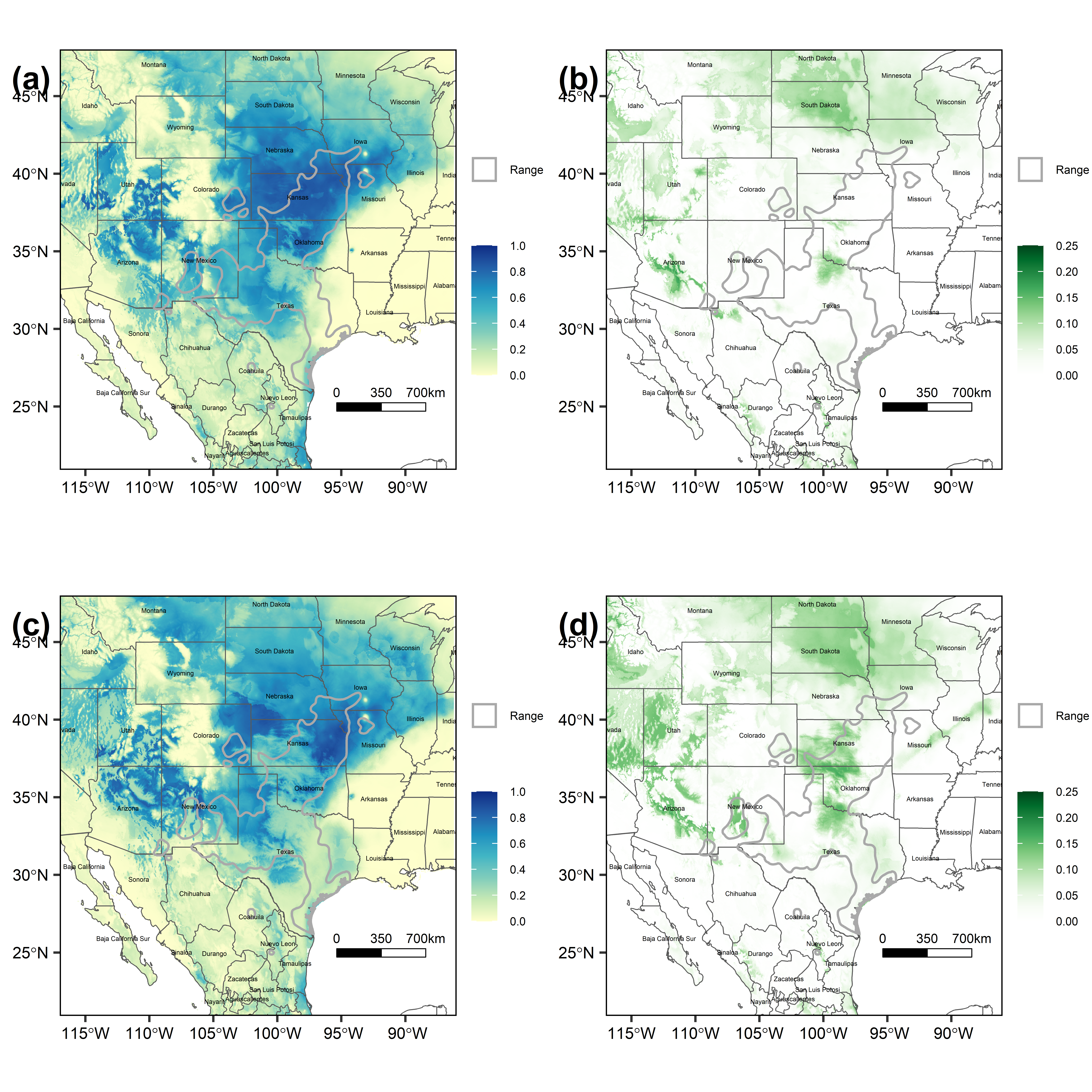


### Figure S1.6. Likelihood of occurrence of *Sistrurus* *tergeminus* forecast under a 2070 2.6 W/m2 warming scenario, estimated from the selected 16 Maxent models. (a) Mean and (b) Variance for the CCSM4 global circulation model (GCM). (c) Mean and (d) Variance for the MIROC-ESM GCM.


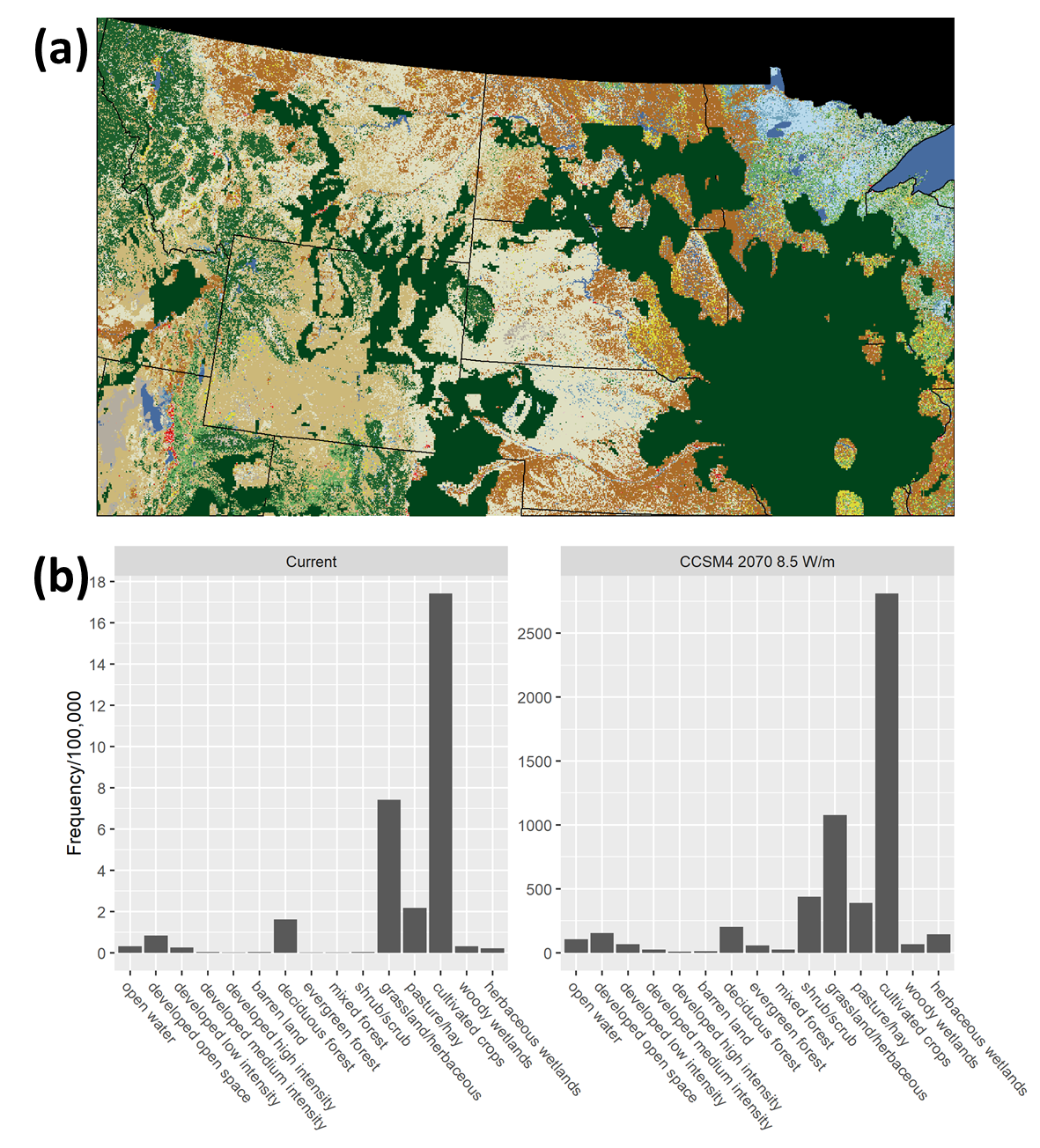


### Figure S1.7. (a) Likelihood of occurrence over 50% in green plotted on the National Land Cover map (Homer, Fry, & Barnes, 2012) under the CCSM4 2070 8.5 W/m GCM. (b) Frequency of land cover classes in the green polygons for the potential shift in climate space into South Dakota, North Dakota, Nebraska, Montana, Wyoming, Iowa, Wisconsin, and Minnesota. This shows that approximately 50% of the space that presents as a suitable climate under the future scenarios may not be available as grassland habitat, as it is already cultivated crops or pasture/hay.

# **References**

Allouche, O., Tsoar, A., & Kadmon, R. (2006). Assessing the accuracy of species distribution models: prevalence, kappa and the true skill statistic (TSS). *Journal of Applied Ecology*, *43*(6), 1223–1232. https://doi.org/10.1111/j.1365-2664.2006.01214.x

Araujo, M. B., Pearson, R. G., Thuiller, W., & Erhard, M. (2005). Validation of species-climate impact models under climate change. *Global Change Biology*, *11*(9), 1504–1513. https://doi.org/10.1111/j.1365-2486.2005.001000.x

Barbet-Massin, M., Jiguet, F., Albert, C. H., & Thuiller, W. (2012). Selecting pseudo-absences for species distribution models: How, where and how many? *Methods in Ecology and Evolution*, *3*(2), 327–338. https://doi.org/10.1111/j.2041-210X.2011.00172.x

Barve, N., Barve, V., Jiménez-Valverde, A., Lira-Noriega, A., Maher, S. P., Peterson, A. T., … Villalobos, F. (2011). The crucial role of the accessible area in ecological niche modeling and species distribution modeling. *Ecological Modelling*, *222*(11), 1810–1819. https://doi.org/10.1016/j.ecolmodel.2011.02.011

Elith, J., & Graham, C. H. (2009). Do they ? How do they ? WHY do they differ ? On finding reasons for differing performances of species distribution models. *Ecography*, *32*(December 2008), 66–77. https://doi.org/10.1111/j.1600-0587.2008.05505.x

Elith, J., Graham, C. H., Anderson, R. P., Dudík, M., Ferrier, S., Guisan, A., … Zimmermann, N. E. (2006). Novel methods improve prediction of species’ distributions from occurrence data. *Ecography*, *29*(2), 129–151. https://doi.org/10.1111/j.2006.0906-7590.04596.x

Hallgren, W., Santana, F., Low-Choy, S., Zhao, Y., & Mackey, B. (2019). Species distribution models can be highly sensitive to algorithm configuration. *Ecological Modelling*, *408*(July), 108719. https://doi.org/10.1016/j.ecolmodel.2019.108719

Homer, C. G., Fry, J. A., & Barnes, C. A. (2012). The National Land Cover Database. In *Fact Sheet*. https://doi.org/10.3133/fs20123020

Jarnevich, C. S., Talbert, M., Morisette, J., Aldridge, C., Brown, C. S., Kumar, S., … Holcombe, T. (2017). Minimizing effects of methodological decisions on interpretation and prediction in species distribution studies: An example with background selection. *Ecological Modelling*, *363*, 48–56. https://doi.org/10.1016/j.ecolmodel.2017.08.017

Landis, J. R., & Koch, G. G. (1977). The Measurement of Observer Agreement for Categorical Data. *Biometrics*, *33*(1), 159–174.

Phillips, S. J., Anderson, R. P., & Schapire, R. E. (2006). Maximum entropy modeling of species geographic distributions. *Ecological Modelling*, *190*, 231–259. https://doi.org/10.1016/j.ecolmodel.2005.03.026

Phillips, S. J., Dudík, M., Elith, J., Graham, C. H., Lehmann, A., Leathwick, J., & Ferrier, S. (2009). Sample selection bias and presence-only distribution models: Implications for background and pseudo-absence data. *Ecological Applications*, *19*(1), 181–197. https://doi.org/10.1890/07-2153.1

Radosavljevic, A., & Anderson, R. P. (2014). Making better Maxent models of species distributions: Complexity, overfitting and evaluation. *Journal of Biogeography*, *41*(4), 629–643. https://doi.org/10.1111/jbi.12227

Roberts, D. R., Bahn, V., Ciuti, S., Boyce, M. S., Elith, J., Guillera-Arroita, G., … Dormann, C. F. (2017). Cross-validation strategies for data with temporal, spatial, hierarchical, or phylogenetic structure. *Ecography*, *40*(8), 913–929. https://doi.org/10.1111/ecog.02881

Ryberg, W. A., Lawing, A. M., & Hibbitts, T. J. (2017). *Habitat Conservation Forecasting for the Western Massasauga (*Sistrurus tergeminus*) in New Mexico*. Final Report, New Mexico Department of Game and Fish, Santa Fe, NM.

Ryberg, W. A, Walkup, D. K., Hibbitts, T. J., Lawing, A. M., DeWoody, J. A., & Bylsma, R. (2020). *Genomics of the Western Massasauga (*Sistrurus tergeminus*)*. Final Report, U.S. Fish and Wildlife Service, Austin, NM.

Shcheglovitova, M., & Anderson, R. P. (2013). Estimating optimal complexity for ecological niche models: A jackknife approach for species with small sample sizes. *Ecological Modelling*, *269*, 9–17. https://doi.org/10.1016/j.ecolmodel.2013.08.011

Sofaer, H. R., Jarnevich, C. S., Pearse, I. S., Smyth, R. L., Auer, S., Cook, G. L., … Hamilton, H. (2019). Development and Delivery of Species Distribution Models to Inform Decision-Making. *BioScience*, *69*(7), 544–557. https://doi.org/10.1093/biosci/biz045

Varela, S., Anderson, R. P., García-Valdés, R., & Fernández-González, F. (2014). Environmental filters reduce the effects of sampling bias and improve predictions of ecological niche models. *Ecography*, *37*(11), 1084–1091. https://doi.org/10.1111/j.1600-0587.2013.00441.x
